# Discovering new pathways toward integration between health and sustainable development goals with natural language processing and network science

**DOI:** 10.1186/s12992-023-00943-8

**Published:** 2023-06-29

**Authors:** Thomas Bryan Smith, Raffaele Vacca, Luca Mantegazza, Ilaria Capua

**Affiliations:** 1 Bureau of Economic and Business Research, University of Florida, nd Ave Ste 150, PO Box 117148, Gainesville, FL 32611 USA; 2 Department of Social and Political Sciences, University of Milan, Milan, Italy; 3 One Health Center of Excellence, IFAS, University of Florida, Gainesville, FL USA; 4 Johns Hopkins University, SAIS Europe, Bologna, Italy

**Keywords:** Sustainable development goals, One health, Natural language processing, Network science, Topic modeling

## Abstract

**Background:**

Research on health and sustainable development is growing at a pace such that conventional literature review methods appear increasingly unable to synthesize all relevant evidence. This paper employs a novel combination of natural language processing (NLP) and network science techniques to address this problem and to answer two questions: (1) how is health thematically interconnected with the Sustainable Development Goals (SDGs) in global science? (2) What specific themes have emerged in research at the intersection between SDG 3 (“Good health and well-being”) and other sustainability goals?

**Methods:**

After a descriptive analysis of the integration between SDGs in twenty years of global science (2001–2020) as indexed by *dimensions.ai*, we analyze abstracts of articles that are simultaneously relevant to SDG 3 and at least one other SDG (N = 27,928). We use the top2vec algorithm to discover topics in this corpus and measure semantic closeness between these topics. We then use network science methods to describe the network of substantive relationships between the topics and identify ‘zipper themes’, actionable domains of research and policy to co-advance health and other sustainability goals simultaneously.

**Results:**

We observe a clear increase in scientific research integrating SDG 3 and other SDGs since 2001, both in absolute and relative terms, especially on topics relevant to interconnections between health and SDGs 2 (“Zero hunger”), 4 (“Quality education”), and 11 (“Sustainable cities and communities”). We distill a network of 197 topics from literature on health and sustainable development, with 19 distinct network communities – areas of growing integration with potential to further bridge health and sustainability science and policy. Literature focused explicitly on the SDGs is highly central in this network, while topical overlaps between SDG 3 and the environmental SDGs (12–15) are under-developed.

**Conclusion:**

Our analysis demonstrates the feasibility and promise of NLP and network science for synthesizing large amounts of health-related scientific literature and for suggesting novel research and policy domains to co-advance multiple SDGs. Many of the ‘zipper themes’ identified by our method resonate with the One Health perspective that human, animal, and plant health are closely interdependent. This and similar perspectives will help meet the challenge of ‘rewiring’ sustainability research to co-advance goals in health and sustainability.

**Supplementary Information:**

The online version contains supplementary material available at 10.1186/s12992-023-00943-8.

## Background

Research on topics related to sustainability and health is growing at an unprecedented pace across multiple disciplines and sectors [1–3]. As a result, scoping reviews, systematic reviews, and bibliometric analyses have become essential tools for synthesizing evidence in this area and designing evidence-based policies [4–7]. However, traditional methods of literature review and synthesis, based on manual expert assessment, increasingly appear too time- and resource-consuming to keep pace with the expanding evidence base and maintain scientists and policymakers abreast of most recent research developments [1–3]. The resulting delays in literature appraisal may withhold insights that could be crucial to the advancement of the United Nations’ (UN) Sustainable Development Goals (SDGs) [8], including the goal to “ensure healthy lives and promote well-being for all at all ages” (SDG 3, “Good health and well-being”) [9]. Scientific appraisal is among the first bottlenecks in the translation of health discoveries into policy and practice. Delays in this stage can have long-term implications for global health and sustainability, especially when societies face global health emergencies such as COVID-19 [10, 11].

Indeed, the health-related, socioeconomic, and environmental impacts of the COVID-19 pandemic have reshaped priorities and underscored the primacy of health for the sustainability of contemporary human societies, spurring calls to prioritize health sustainability and shift towards a One Health perspective in science and policy [12, 13]. Since its very early stages, the COVID-19 emergency has caused a significant setback for the world’s advancement toward sustainable development, especially among the poorest countries and most vulnerable social groups [14]. As the initial emergency has subsided, the health inequalities [15–17], socioeconomic determinants of health [18, 19], and environmental challenges [20, 21] exposed by the pandemic have led to the reevaluation of the health-focused SDG 3 as a central goal capable of guiding holistic and coherent policies for sustainability and prompting synergistic actions in favor of multiple other sustainability goals [22]. The kind of sustainability synergies that SDG 3 can promote may occur through second order effects – e.g., when improving the health of the working population (SDG 3) also improves the state of the economy (SDG 8) – or by requiring explicit advancement of another goal – e.g., reducing the spread of waterborne diseases (SDG 3) by improving sanitation (SDG 6) [23].

The COVID-19 pandemic has also highlighted the complex networks of connections and interdependencies between the 17 SDGs. Development goals and their targets form an interconnected and dynamic system, with numerous and varying synergies and trade-offs among them [24–26]. The same scientific insight or policy action may co-advance multiple SDGs simultaneously or contribute to one while hindering others. This creates a need to map SDG interdependencies over time and to ensure policy coherence in sustainability – the harmonization of policies to simultaneously address multiple SDGs, optimize resources for SDG co-advancement, and prevent harms to public health from underinformed policymaking [27–29].

Against this backdrop, this paper analyzes the body of global scientific research that has addressed the health-related SDG 3 in the past twenty years, and its potential to promote synergistic progress, in science and policy, on multiple other SDGs simultaneously. Specifically, we seek to answer the following research questions: (1) How is SDG 3 thematically interconnected with all other SDGs in global scientific research, and how have these interconnections changed since the turn of the millennium? (2) What specific themes and topics have emerged in research conducted at the intersection between SDG 3 and other SDGs in the last twenty years, and how can we identify the topics that are most useful to fuel progress towards multiple SDGs simultaneously? With the first question, we explore the extent to which global science has addressed topics that are relevant to both SDG 3 and other SDGs, identifying which other SDGs are consistently overlapping with research on global health and health sustainability, and how this has changed over the past twenty years. Considering the second question, we map the themes and topics that have emerged in global science around SDG 3 and other SDGs, and the network of substantive, semantic interrelationships underpinning them.

Addressing these two questions, this study makes two important contributions to research on global health, public health, sustainability, and globalization. First, we advance a novel approach to the study of interdependencies between SDGs, in which large amounts of research outputs are analyzed with Natural Language Processing techniques and network science methods to synthesize knowledge, map links among SDGs, and describe entry points for science and policy to co-advance multiple SDGs simultaneously. We apply this approach, specifically, to the interdependencies between the health-related SDG 3 and all other SDGs. Second, we identify ‘zipper themes’ in science and policy around health and sustainability. Zipper themes are topics, scientific questions or policy issues which can strengthen scientific research and suggest synergistic policies for co-advancing SDG 3 together with other sustainability goals. Each SDG is itself a broad set of themes and objectives formulated to facilitate communication and collaboration between scientists and policymakers from a wide variety of fields, policy areas, and agendas – from energy and economy to health and biodiversity. We identify more granular zipper themes – within and across SDGs – that will stimulate innovative research ideas around the health-sustainability nexus, promote a conceptual and terminological convergence in sustainability research, and guide the formulation of coherent and actionable policies to co-advance goals in multiple sustainability areas.

### COVID-19, health sustainability, and SDG interdependencies

Originally conceived at the Rio + 20 conference as part of the UN’s 2023 Agenda, the SDGs are a set of broad aims, specific targets, and related indicators concerning social, political, economic, and environmental sustainability and aiming to promote global cooperation for sustainable societies [8]. The third SDG, the main focus of this paper, is labelled as “Good health and well-being” and aims to “Ensure healthy lives and promote well-being for all at all ages” by 2030: its targets include reducing the global maternal mortality ratio; ending the epidemics of AIDS, tuberculosis and malaria; achieving universal health coverage; substantially reducing the prevalence of deaths and illnesses from pollution and chemical contamination; and strengthening the capacity of developing countries for management of national and global health risks.

SDG 3 is clearly linked to other SDGs, in the sense that – given the crucial health impacts of certain social, economic, and environmental factors – progress towards SDG 3 clearly goes hand in hand with progress towards other goals, such as SDG 2 (Zero hunger: End hunger, achieve food security and improved nutrition and promote sustainable agriculture) or SDG 13 (Climate action: Take urgent action to combat climate change and its impacts). Indeed, the SDG targets and indicators are inherently interconnected by relationships of synergy, when certain actions can contribute to multiple goals at the same time; and trade-offs, when actions advancing one goal can inhibit or harm progress on another [25, 30]. This network of interdependencies is not static over time and space, but can change depending on geography (e.g., in high-income vis-à-vis low-income countries) and time [26, 29]. Mapping and evaluating SDG interdependencies is essential to ensure coordination and coherence of policies towards sustainable development at the global, national, and regional levels [28]. Previous research has quantified these SDG interconnections by analyzing co-variance among SDG or target indicators over time 26, 31, 32; or by surveying experts to qualitatively distill synergies and trade-offs from previous scientific literature [33–35]. While these efforts have been insightful, their approaches are constrained by cost and at times limited availability of indicator data, or by the difficulty of assessing exponentially growing volumes of literature in reasonable time via qualitative, manual expert appraisal – especially when SDG synergies and trade-offs must be evaluated at different geographic locations or scales, or during crises requiring rapid intervention. Overcoming these constraints is important for the effective and time-sensitive translation of scientific discoveries into practice, interventions and policies for health and sustainability, particularly in times of environmental or health crises [11]. In this light, new computational methods, leveraging recent advances in machine learning and natural language processing, can complement existing ones and address some of their drawbacks [36].

In the last three years, the complex network of SDG synergies and trade-offs has been brought into sharp focus by COVID-19. The first-order effects of the pandemic on public health revealed that many countries had significant room for improvement with respect to SDG 3, especially in terms of resilience of their health systems to crises [14, 37]. With its second-order effects in the social, economic, and environmental domains, the COVID-19 crisis negatively impacted indicators of poverty (SDG 1), education (SDG 4), and unemployment (SDG 8), while it brought about a short-term reduction in greenhouse gas emissions, positively impacting climate action (SDG 13) [37]. Further exposing interconnections between different sustainability goals, COVID-19 has also emphasized the urgency of adopting a One Health perspective on issues of health and sustainability [13]. This perspective posits that the health of humans, domestic and wild animals, plants, and the environment are strictly interconnected by co-benefits and trade-offs [12, 13, 38]. Exemplifying the link between human and animal health, COVID-19 itself has been classified as a zoonotic disease due to the genetic similarities between SARS-CoV-2 and horseshoe bat coronaviruses, while natural resource consumption (relevant to SDG 12) and climate change (the focus of SDG 13) have been indicated as causes of increased rates of interaction and potential pathogen transmission between species [12]. At the same time, recent research has highlighted how the advancement of SDGs 12 (Responsible consumption and production) and 13 (Climate action) can directly influence SDG 3, both positively via improvements in air quality and other environmental determinants of health, *and* negatively via socio-economic trade-offs that reduce pollution and consumption, such as unemployment caused by the shuttering of coal-fired plants in nations without universal health care [23]. Together with a commitment to ‘health in all policies’ by the United States Center for Disease Control and Prevention [39], some of the most vocal proponents of a One Health perspective have recently been the G7, G20, Global Health Summit, and World Health Organization [40–42].

Finally, the pandemic has also forced rapid innovations in data analysis [43]. While these innovations have generally been focused on the monitoring of health indicators and epidemic modeling, Natural Language Processing (NLP) has also entered the foreground with hundreds of studies seeking to analyze the fast-evolving scientific literature on COVID-19 for information retrieval and summarization, literature-based discovery, question answering, and topic modelling [36]. NLP is a subfield of computational linguistics working towards the goal of developing and ‘training’ machine learning algorithms that can ‘understand’ and unpack the nuances of human speech and written text, able to retrieve syntactic patterns and dependencies in human writing, distill key words, phrases, entities and topics from large amounts of text, and quantify similarities between documents. Sustainability scholars have begun fine-tuning these algorithms to summarize extensive and evolving bodies of sustainability-related scholarship [3, 44]. For example, addressing SDG 2 (Zero hunger), Porciello et al. (2020) recommended the wider application of NLP for mapping similarities between texts, entity-recognition, and coreference resolution, with the goal of accelerating the synthesis of large quantities of evidence in sustainability, and thereby efficiently discovering effective policies and practices for sustainable development [44]. Focusing on the climate-related SDG 13, Callaghan et al. (2021) fine-tuned the DistilBERT language model to categorize and extract specific information from 102,160 climate impact studies. They used the results to map field-wide trends in anthropogenic climate change (1951–2018) [3]. In these studies – as well as in other works of automated, NLP-based synthesis of evidence which became popular during the pandemic [36] – the goal is to examine specific bodies of literature, providing a highly detailed treatment of a single SDG domain or topic. In contrast, we broaden the focus to relationships among multiple SDG domains and propose a method which combines NLP and network science techniques to illuminate interdependencies between SDGs and generate insights co-advancing multiple sustainability goals together.

## Methods

The methods of this study consist of two steps. First, using results from an existing machine learning method to classify scientific publications by their SDG relevance [45], we determine the frequency with which scientific research has addressed the health-related SDG 3 and each other SDG over the past twenty years, identifying SDGs that are well or poorly integrated with SDG 3 in global science. Second, we implement a method, based on topic modeling and network science, to zoom in on the actual contents of scientific research at the intersection between SDG 3 and other SDGs, to map and describe substantive themes of convergence and overlap. All analyses and visualizations were performed using the top2vec library in the Python general-purpose programming language, and the igraph and CentiServer packages in the R statistical computing software within the Visual Studio Code IDE [46–50].

### Data

We analyze all peer-reviewed scientific articles, published between 2001 and 2020, which are indexed as relevant to SDG 3 and one or more other SDGs in Dimensions, the most exhaustive database for scientific publications [51, 52]. Data about titles and abstracts of these articles were collected using custom built functions that create an interface between the R statistical computing environment and the Dimensions API. Each article’s relevance to each of the 17 SDGs is determined by a classification algorithm developed by Dimensions and returning a binary index of relevance to each SDG: i.e., an index classifying an article as either relevant or not relevant to each of the 17 SDGs [45]. Created by Digital Science et al. (2020), the classification algorithm was trained on a data set consisting of articles that are certainly relevant to each SDG. These articles were found with a specific keyword search query, for each SDG, of works published since 2010 (when the Millennium Development Goals, the SDGs’ precursors, were established). The keyword search queries were manually curated and informed by the UN’s SDG definitions, targets, and indicators, aiming to minimize false positive rates. Supervised NLP algorithms were then trained to make binary classification decisions (relevant/not relevant) based on the training data corresponding to each of the 17 SDGs. In the results, each article may be classified as relevant to one, multiple, or none of the 17 SDGs. The articles selected for our data were classified as relevant to SDG 3 and at least one other SDG. These are 27,928 articles, contributed by 75,665 authors. Our text data is limited to their abstracts, which overall contain 3,918,143 tokens (units of speech, e.g. words) and 64,575 unique tokens (of which 36,550 appear more than once in the corpus). Procedures for preprocessing this corpus of text are detailed in the supplementary materials.

### Topics, topic networks and communities

To distill topics in the article abstracts we employ top2vec [49], a recent unsupervised machine learning approach to discovering topics in large text corpora via word and document embeddings, implemented in the *top2vec* Python library. This method combines the word2vec and doc2vec embedding models [53, 54], Uniform Manifold Approximation and Projection for dimension reduction (UMAP) [55], and hierarchical density-based spatial clustering of applications with noise (HDBSCAN) [56]. The word2vec model uses shallow neural networks to learn numeric representations (i.e., vectors or embeddings) of words based on collocation with other words in text. Relationships between these numeric representations approximate human understanding of semantic relationships between words; semantically related words like virus, vaccine, and epidemic appear in similar contexts and are therefore represented by similar numbers, and semantically dissimilar words like virus and volcano will be represented by different numbers [53]. The doc2vec model inherits word2vec’s understanding of semantics and adds to it by concurrently learning numeric representations of documents (here, article abstracts), in addition to words [54]. The resulting embeddings, which provide a numeric representation of each word and abstract in the corpus, are numeric vectors of 300 dimensions, that is, each including 300 numbers. The third component of the method, UMAP, reduces these high-dimensional embeddings onto fewer dimensions (fewer numbers): that is, it projects the embeddings in low-dimensional space. Finally HDBSCAN is used to identify dense clusters of documents that have similar embeddings in this low-dimensional space [49]. In the results, a cluster of documents (i.e., of scientific articles) represents a distinct substantive topic; and the centroid of the document embeddings in that cluster (a sort of “average” embedding of the cluster) provides a numeric representation of that entire topic (i.e., the topic embedding). Applied to our corpus of articles, the top2vec method detects a total of 197 topics (clusters). These are summarized in Table S2, including the number of articles in each topic (from 26 articles in the smallest topic to 1277 in the largest) and a description of topic contents based on the most salient words in the topic (words with embeddings that are closest to the cluster centroid).

We quantify semantic closeness between two topics by measuring similarity between their respective topic embeddings via cosine similarity, a popular measure of similarity between two numeric vectors. The result is a network uncovering the structure of semantic relationships between topics (see Fig. 3): each network node is a topic, and the weighted link between two nodes represents the semantic closeness between two topics (i.e., the cosine similarity between their embeddings). Considering breaks in the distribution of cosine similarity scores, a global edge filter is set on this weighted network to only retain links between two topics when their cosine similarity is higher than the 99th percentile (cosine similarity ≥ 0.279). The result is a network in which two topics are linked if they are semantically related or not linked if they are not related, and the precise cosine similarity scores between two topics can, if necessary, be disregarded. Like many social and semantic networks [57], this topic network has a “community structure”: it consists of different communities, that is, distinct groups of topics (nodes) that are more closely connected to each other (i.e., more semantically similar to each other) and more distant from all other nodes. We identify these communities using the walktrap community detection algorithm (from the R igraph package, setting the number of algorithm steps to 5). [48, 58] This results in 19 communities – each gathering between 2 and 30 topics (Tables S1 and S2) – which reveal broad yet coherent thematic clusters in scientific research around SDG 3 and other SDGs in 2001–2020.

We also use UMAP again (from the *umap-learn* Python library 55) to visualize the distribution of SDGs across articles on a 2-dimensional plane – a ‘topic map’ of research around health and sustainability (Fig. 2). Each point in this map is an article, and two articles are close in space when they are topically, semantically, or lexically similar (and distant when they are dissimilar). Points (articles) are colored in accordance with the second SDG they were assigned by the Dimensions classification algorithm, in addition to SDG 3-Health (light grey points are articles assigned more than one other SDG). The approximate regions corresponding to each SDG in this topic map, as well as the overlaps between these regions, are visualized in Figure S3.

### Identifying zipper themes

The network representation of the semantic relationships among topics allows the identification of zipper themes for co-advancing health and other SDGs in science and policy. We propose three methods to distill zipper themes from the topic network: network centrality, cross-community connections, and isolates. First, *network centrality* measures (from the *CentiServer* R package 50) detect topics that occupy more central positions in the network’s structure of connections. These topics can be regarded as zipper themes: their high centrality indicates that they are relevant or connected to many other topics at the interface between health and sustainability [59–61]. Specifically, we use betweenness and harmonic closeness measures of centrality, as well as the Density of the Maximum Neighborhood Component (DMNC) [62–64]. Detailed descriptions of these centrality measures are provided in the supplemental materials. Second, *cross-community connections* are links between topics (nodes) that belong to separate communities in the network. Recall that 19 distinct communities of topics are discerned in the network. Connections between nodes in different communities reflect semantic relationships between topics in areas that are otherwise distant in sustainability and health research. Thus, cross-community connections point to existing zipper themes (links of semantic closeness that already exist in the current network) that bridge different research areas in the field. Finally, a small selection of 12 topics discovered by top2vec were separated from all other nodes after imposing the global filter, becoming *isolates*. The articles forming these isolated topics are substantively or discursively distinct from the rest of the corpus even though (by construction of the data) they are still classified as relevant to SDG 3 and at least another SDG. Hence, these isolates indicate gaps in the network structure – and point to corresponding gaps in science and policy on health and sustainability: topics that are semantically distant from all others in the current network, but that further research or interventions could bridge with the “mainstream” of the health and sustainability knowledge network.

## Results

### Mapping interconnections between SDG 3 and other SDGs in global research

**Fig. 1:**
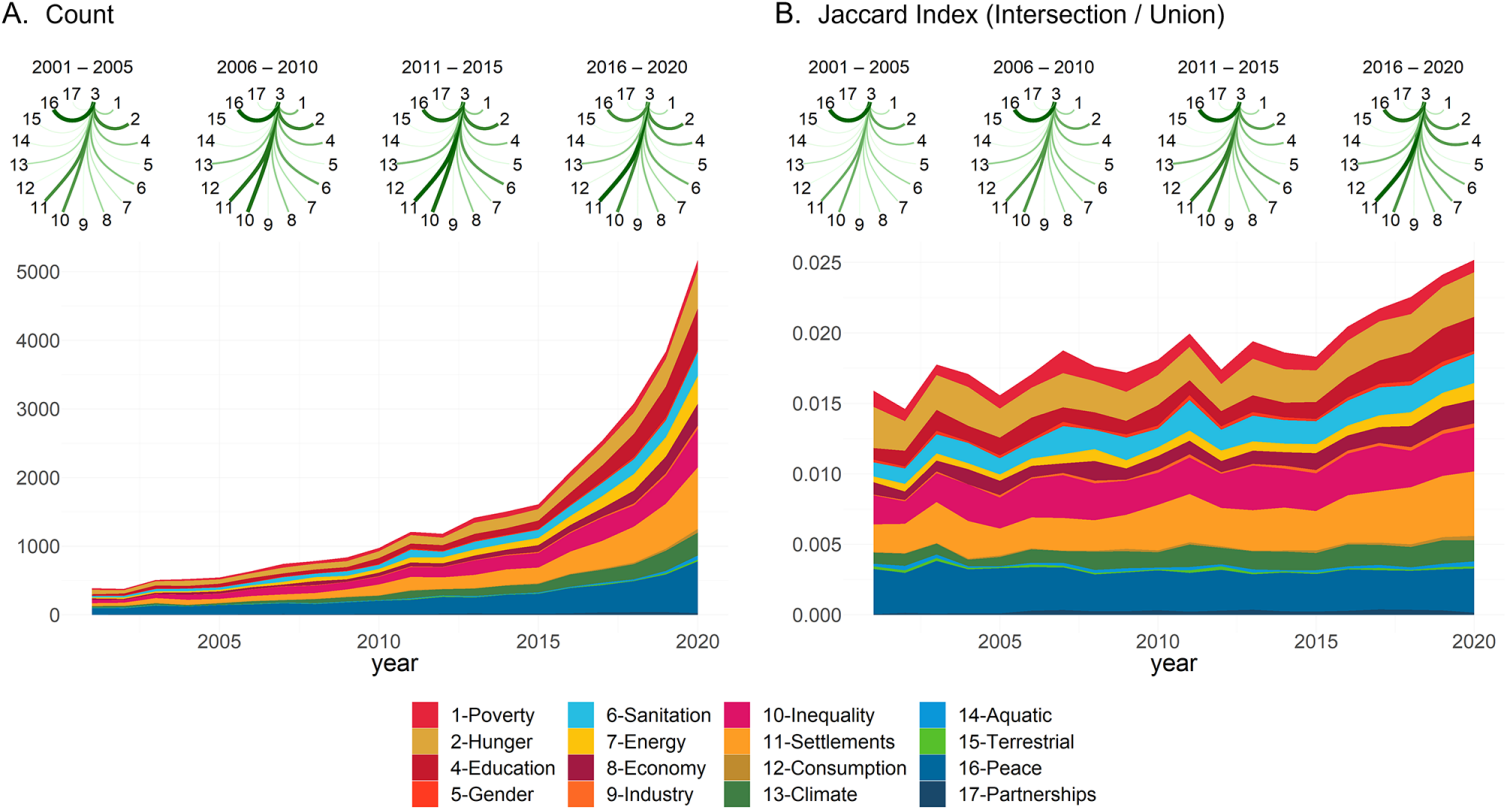
Intersection of scientific research relevant to SDG 3-Health with research relevant to each other SDG. (**A**) Raw count of scientific articles classified as relevant to SDG 3 and another SDG. (**B**) Jaccard index of the set similarity between articles relevant to SDG 3 and those relevant to each other SDG. All articles in the corpus are classified by dimensions.ai as relevant to SDG 3 and at least another SDG.

Figure 1 visualizes the overlap between SDG 3-Health and all other SDGs in relevant scientific literature between 2001 and 2020. Scientific research on SDG 3 most frequently intersects with literature on SDGs 16-Peace (N_pub_ = 5,167), 11-Settlements (N_pub_ = 4,628), 10-Inequality (N_pub_ = 3,610), 2-Hunger (N_pub_ = 3,243), and 4-Education (N_pub_ = 2,764). In contrast, SDGs with the smallest intersecting literature with SDG 3 (less than 300 articles across all years) are 15-Terrestrial, 12-Consumption, 9-Industry, 14-Aquatic, and 17-Partnerships. Counts of publications relevant to SDG 3 and another goal have increased markedly in the last 5 to 10 years (+ 139% in 2015–2020, + 74% in 2010-15), most notably for 11-Settlements (+ 176% publications shared with SDG 3 in 2015–2020, N_2020_ = 1,791), 4-Education (+ 274% publications in the same period, N_2020_ = 1,314), and 16-Peace (+ 101% publications, N_2020_ = 1,268). The intersection between SDG 3 and other SDGs in global science has also grown as a proportion of the overall size of literature addressing any pair of SDGs (Fig. 1.B), particularly in the last 5 years, with SDGs 11-Settlements, 4-Education and 2-Hunger showing the highest standardized overlap (Jaccard index) with 3-Health.

**Fig. 2:**
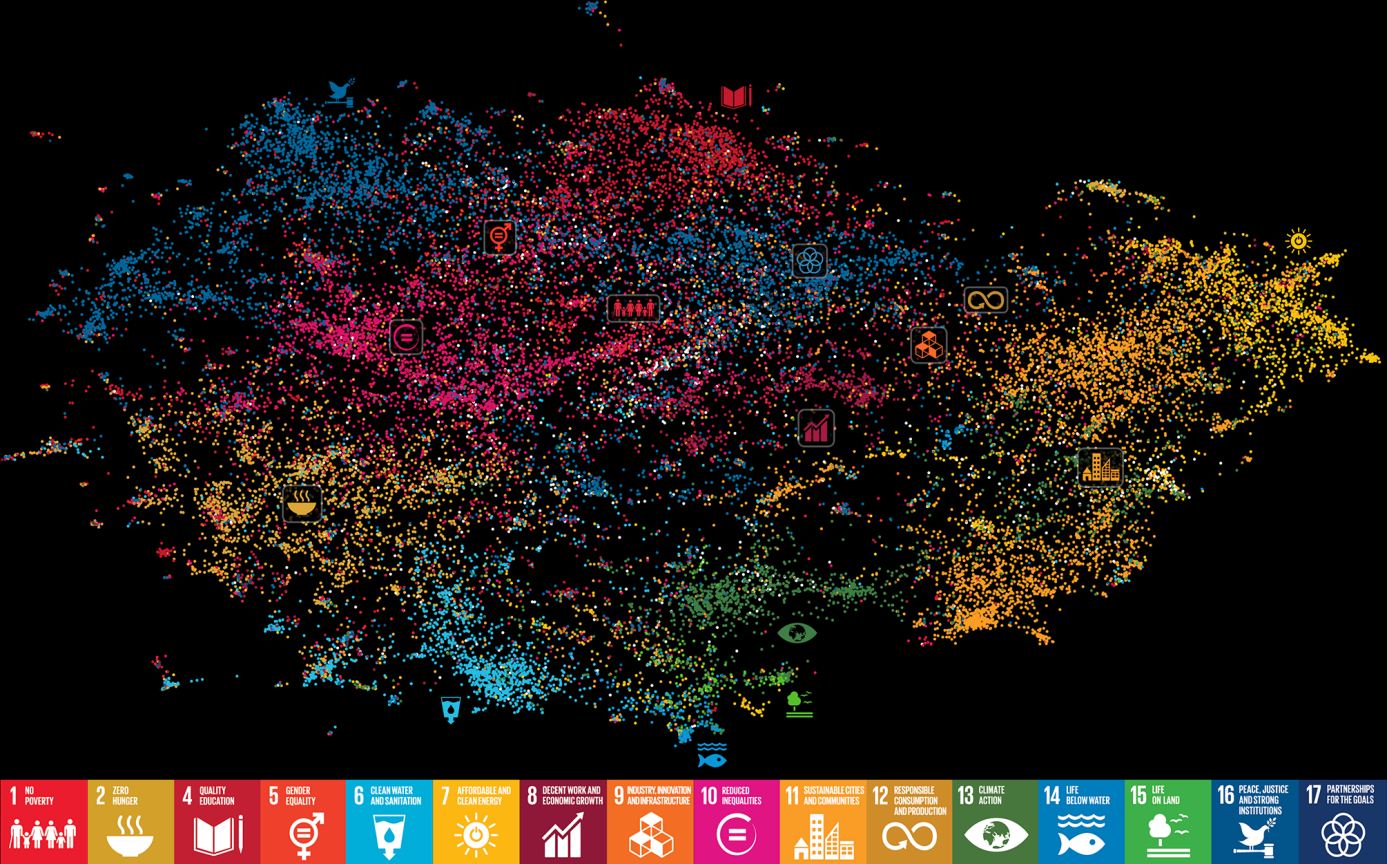
Topic map of all peer-reviewed publications classified by dimensions.ai as relevant to SDG 3-Health and at least one other SDG. Each point is a publication, proximity between two points indicates topical or semantic proximity between the two corresponding publications in a UMAP 2D projection of document embeddings (see Methods). Point colors indicate the secondary SDGs (other than 3-Health) to which each article is relevant. Publications classified as relevant to three or more SDGs are in light grey

Figure 2 presents a topic map of scientific research relevant to SDG 3-Health and at least another SDG, based on the same corpus of articles as in Fig. 1. A strong divide is observed between SDGs in the socioeconomic domain (top-left region of the map: 1-Poverty, 2-Hunger, 4-Education, 5-Gender, 8-Economy, 10-Inequality, 16-Peace, 17-Partnerships) and environmental SDGs (bottom-right region: 7-Energy, 11-Settlements, 13-Climate, 14-Aquatic, 15-Terrestrial). However, the map also provides clues about important points of integration between the socioeconomic and environmental goals: literature relevant to SDG 6-Sanitation lies at the intersection between 2-Hunger and {14-Aquatic, 15-Terrestrial} in the map; articles on 9-Industry and 12-Consumption are situated at the intersection of those related to {8-Economy, 17-Partnerships} and {7-Energy, 11-Settlements}; the last two are also close to 13-Climate, 14-Aquatic, and 15-Terrestrial. These proximities in the map – and the adjacency between articles attached to SDGs in the same broad area (e.g., SDGs 5 and 10 on social inequalities, or the environmental SDGs 13, 14 and 15) – suggest that the method we used to distill topics and topical proximities in SDG-related science captures meaningful themes and relationships in the text corpus. This allows us, in the next section, to narrow the focus to substantive themes of overlap between research on different SDGs.

### Topics and zipper themes at the health-sustainability nexus

**Fig. 3:**
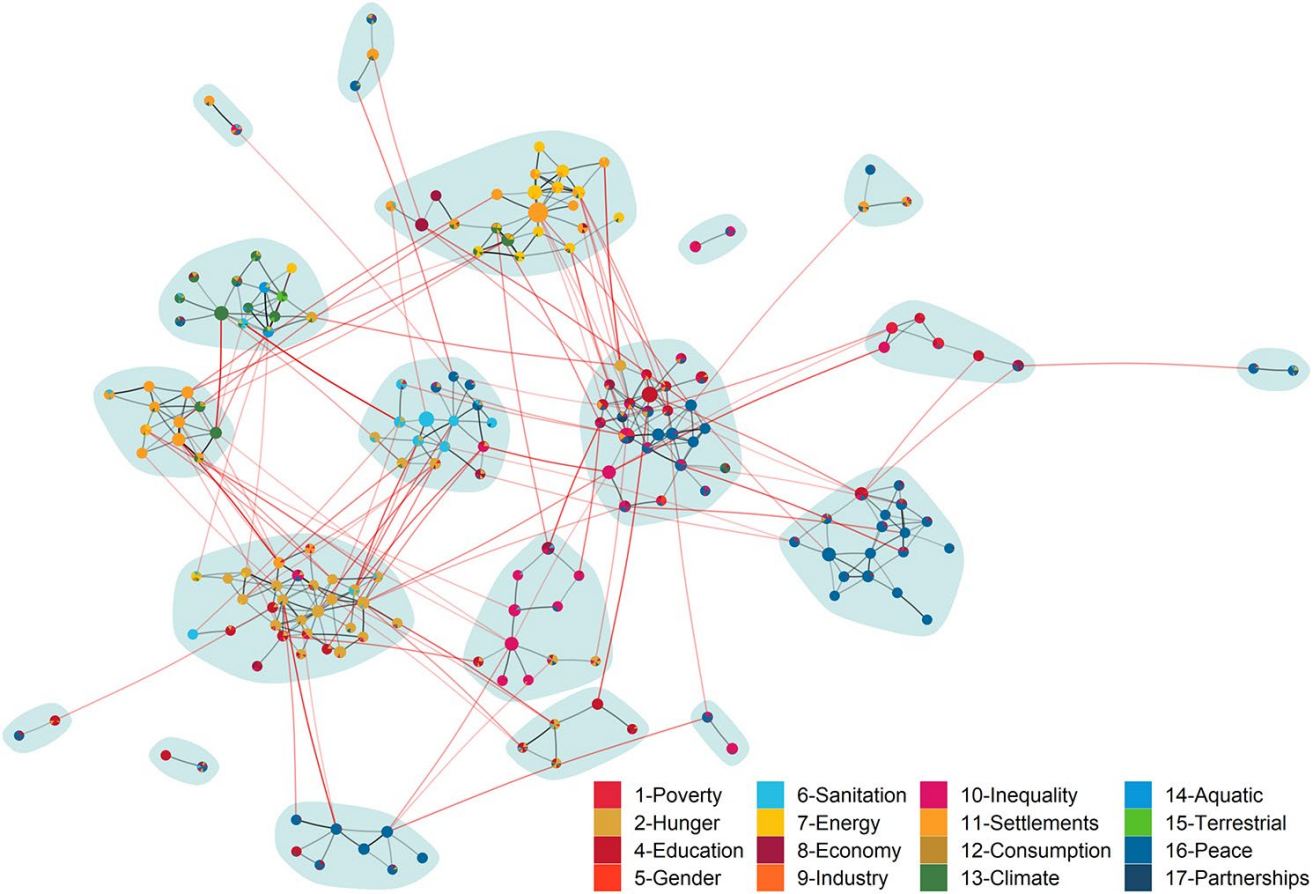
Semantic network of topics in science related to SDG 3-Health and at least another SDG. Nodes are top2vec topics, drawn as pie charts whose colors represent the SDGs to which publications in each node (topic) are relevant, according to dimensions.ai classification. Node size represents harmonic closeness. Weighted edges are cosine similarities between the embeddings of two topics (when similarity > 99th percentile). Light-blue polygons are network communities: within-community edges are black, between-community edges are red. Network isolates are removed

Figure 3 shows the network of semantic connections between topics in scientific research around SDG 3-Health and other SDGs. *Highly central topics* in the network are semantically connected and potentially relevant to a great number of other topics, are part of large and dense thematic regions, or bridge separate areas in research about SDG 3-Health and other sustainability goals. A consistent set of highly central topics emerges across all centrality measures, producing a stable ‘core’ of zipper themes according to this criterion (e.g., topics 1, 2, 8, 9, 20 in Figure S4 and Table S2). Substantial correlations are observed between network centrality of topics and counts of publications in each topic (from 0.16 for DMNC to 0.39 for closeness and 0.58 for betweenness centrality), suggesting that central themes are also relatively well-established in science, catalyzing larger amounts of research. Topics 2 (health-relevant targets ioutlined by the Millennium and Sustainable Development Goals) and 9 (reproductive health and healthcare, access to skilled birth attendants, maternal mortality rates in developing countries) are among the most central in this analysis. Notably, the publications in topic 9 (e.g., Ali & Chauhan 2020; Pulok et al. 2016 in the bibliography, see also Table 1) [65, 66] tend to explicitly discuss reproductive health in the context of the millennium and sustainable development goals, a topic that is directly relevant to the first target of SDG 3. Additionally, central topics 1, 8, and 20 all consist of research relevant to specific targets and indicators related to health and different SDGs: public transportation and pollution in developing urban environments for topic 1 (e.g., Di Mascio et al. 2018; Jacyna et al. 2017) 67, 68; health inequalities in developed countries for topic 8 (e.g., Leclerc et al. 2006; Stringhini et al. 2015) 69, 70; malnutrition and child mortality in developing countries for topic 20 (e.g., Roy et al. 2018; Tariku et al. 2016) [71, 72]. These substantive contents validate our approach to literature synthesis and identification of zipper themes; they also reinforce the notion of the SDGs as a set of unifying concepts and targets within health-sustainability research.

**Table 1:** List of example articles discussed in reference to topics of interest. Articles are categorized by method of identification (network centrality, cross-community pairing, or network isolation). Constituent topics are indicated and described

Method	Topic: *Description*	Example Article(s)
Centrality	**1**: *Public transportation and pollution in developing urban environments*	Di Mascio et al. (2018) [67] Jacyna et al. (2017) [68]
Centrality	**8**: *Health inequalities in developed countries*	Leclerc et al. (2006) [70] Stringhini et al. (2015) [69]
Centrality	**12**: *Health and safety in the workplace and occupational injuries*	Yilmaz et al. (2016) [73] García-Mainar & Montuenga-Gómez (2009) [74]
Centrality	**20**: *Child mortality in developing countries*	Roy et al. (2018) [71] Tariku et al. (2016) [72]
Cross-community	**9**: C*auses of inequality in maternal healthcare utilization*	Ali & Chauhan (2020) [65] Pulok et al. (2016) [66]
	**68**: *Unequal childhood vaccination coverage*	Hajizadeh (2018) [75] Bobo & Hayen (2020) [76]
Cross-community	**42**: *Schistosomiasis treatment*	Poggensee et al. (2005) [77] Siza et al. (2015) [78] M’Bra et al. (2018) [79]
	**158**: C*limate change, habitat loss, freshwater snail migration, and schistosomiasis*	Pedersen et al. (2014a) [80] Pedersen et al. (2014b) [81]
Isolate	**79**: H*azards, accidents and occupational health in oil tankers and the maritime transportation industry*	Eliopoulou et al. (2012) [84] Uğurlu et al. (2015) [85]
Isolate	**173**: *Advances in research on the children of HIV/AIDS patients and victims*	Short & Goldberg (2015) [82] Wete et al. (2019) [83]

DMNC performs well as a tool for identifying topics at the intersection of wider themes in the corpus (see Table 1 and Figure S5.B). Topic 12 – health and safety in the workplace and occupational injuries (e.g., Yilmaz et al. 2016; García-Mainar & Montuenga-Gómez 2009) [73, 74] – scores the highest on this metric, closely followed by seven topics covering a range of issues, from barriers to healthcare (topic 10) to the health impacts of pollution and climate change (topic 114). Topic 12 is at the intersection of three network communities: itself assigned to a community on the relationship between technology and health (community 3 in Table S1), it is immediately adjacent to communities broadly addressing issues of health emergency response, and health policies, politics, and ethics (communities 11 and 8, respectively). For example, topic 12 is connected to topic 25 (natural disaster injury and response) in community 11, and to topics 102 (micro-entrepreneurship and small-to-medium business development) and 109 (use of communications technology to reduce health care inequalities) in community 8.

*Cross-community links* point to several strong, actionable areas of integration between disparate research on health and sustainability. These areas include both cases in which the same overall issue is treated from two different perspectives, and cases in which two entirely different issues are linked by a third common topic. For example, the connected topics 9 (causes of inequality in maternal healthcare utilization) and 68 (unequal childhood vaccination coverage, e.g., Hajizadeh 2018; Bobo & Hayen 2020) [75, 76], are both part of a broader literature about maternal and infant healthcare (Table 1). However, topic 9 is assigned to a network community which focuses primarily on policies, politics, and ethics, where topic 68 is assigned to a community focused primarily on infectious diseases. Another set of cross-community connections are observed between topics on air pollution (topics 11, 25, 36) and non-communicable diseases among infants and pregnant women (topics 49, 48), adults (topics 83,178), and the elderly (topic 71). The cross-community link criterion also points to an interesting area of overlap between SDGs 13-Climate, 14-Aquatic, and 3-Health: the link between topics 42 (schistosomiasis treatment, a topic in community 9: infectious diseases) and 158 (schistosomiasis infection due to changes in freshwater snail habitats caused by climate change and infrastructure projects, a topic in community 10: wildlife ecology). One body of research investigates prevalence, impact, and treatment of schistosomiasis (e.g., Poggensee et al. 2005; Siza et al. 2015; M’Bra et al. 2018) [77–79], a neglected tropical disease caused by Schistosoma worms; while the other examines its zoonotic etiology (e.g., Pedersen et al. 2014a; 2014b) [80, 81]. Together, these separate but related research agendas offer an ideal example of zipper theme relevant to the One Health perspective.

The strongest cross-community edges (with cosine similarities 2 standard deviations greater than the mean) are between topics 42—158 (cos = 0.59), 6—27 (cos = 0.56), and 11—58 (cos = 0.53). Topics 42 and 158 were described above. Topics 6 (in community 10: wildlife ecology) and 27 (in community 12: environmental health) both address direct impacts of climate change on health: the former focuses on how rising temperatures result in the spread of arboviruses into temperate environments, and the latter on heat waves and heat-related deaths in traditionally temperate climates. Finally, topics 11 (in community 12: environmental health) and 58 (in community 6: non-communicable diseases) both encompass publications on the effects of ambient air pollution, with focuses on respiratory and cardiovascular health, and reproductive health, respectively. These topics are part of a broader area of research into air pollution and the etiology of non-communicable diseases.

The third proposed method to identify zipper themes on health and sustainability – examination of *network isolates* – highlights a disparate set of topics, such as the impact of HIV/AIDS on patients’ children and orphans (topic 173); CRISPR-Cas9 genome editing, the development of gene-edited pathogen-resistant crops, and CRISPR-Cas9 vaccines (topic 90); shipping industry and maritime logistics, occupational safety in this industry, industry-related pollution (e.g. due to oil spills), ecotoxicology and damage to marine organisms (topic 79). These disconnected topics point to research directions with the potential to span significant gaps in existing literature on health and sustainability.

For example, in topic 173, advances in research on the children of HIV/AIDS patients and victims (e.g., Short & Goldberg 2015; Wete et al. 2019) [82, 83] – including immediate medical consequences of parents’ illness on children’s health (e.g., HIV/AIDS infection) and on long-term trajectories of child development and mental health – could have important impacts on a wide range of scientific topics at the intersection between SDG 3 and other sustainability issues. This theme connects at least three topical communities emerging in our synthesis of literature: non-communicable diseases and conditions (community 6); health policies, politics, and ethics (community 8); infectious diseases (community 9). In topic 79, hazards, accidents and occupational health in oil tankers and the maritime transportation industry have impacts both on the health of workers in that sector (for example, ship crews) and on local environments (e.g., Eliopoulou et al. 2012; Uğurlu et al. 2015) [84, 85]. Accidents and disasters in this sector can also have long-term effects on the health of residents in surrounding areas, underscoring significant overlap with research on non-communicable diseases and conditions (i.e., community 6) [86]. This theme is potentially relevant to three topical communities in our literature synthesis: technology, infrastructure, and workplace safety (community 3); wildlife ecology and zoonoses (community 10); and environmental health (community 12).

## Discussion

This study used Natural Language Processing and network science methods to synthesize the entire corpus of scientific abstracts published in 2001–2020 on topics related to SDG 3 (Good health and well-being) and one or more other SDGs. This synthesis was motivated by two main research questions, corresponding with two aims. First, it sought to describe the degree and nature of integration between research relevant to SDG 3 and to other SDGs in global scientific literature over time. Second, it aimed to identify sets of topics, scientific questions, or policy issues – dubbed here as ‘zipper themes’ – which have the potential of stimulating convergence and synergy between research and policy efforts to simultaneously co-advance health (SDG 3) and other sustainability goals.

Addressing the first question and aim, we observed increasing integration between SDG 3 and most of the other goals. This growing body of inter-SDG literature underscores the need for literature reviews that focus on important points of intersection and convergence between goals. While studies on the synergies and trade-offs between all SDG targets and indicators is essential [25, 26, 34], these broader efforts often fail to identify existing or emerging research topics with the potential for a translational impact on the 2030 agenda. The methods presented here can help direct and supplement studies whose aim is to scrutinize overlaps between specific goals, targets, or indicators in an effort to suggest synergistic policies and practices (e.g., De Neve & Sachs 2020) [23].

Scientific integration between SDG 3 and other goals is especially developed with SDG 16-Peace (on topics such as correctional population health, bioethics, and patenting and trade of health technology: topics 7, 19, and 21 in Table S2, respectively); SDG 11-Settlements (for example, on sustainable inner city transport and air pollution: topics 1 and 25); SDG 10-Inequality (on socioeconomic health disparities and ante/postnatal care: topics 8 and 9); SDG 2-Hunger (on food insecurity in HIV-positive populations and HIV-Exposed Uninfected infants, growth disorders and stunting resulting from poor nutrition, sugar intake and obesity: topics 17, 20, 22); and SDG 4-Education (on postgraduate education in health and healthcare: topic 3). Research in these areas is producing scientific knowledge that can help co-advance multiple sustainability goals. The interconnections between SDGs 3, 11, and 16 found in this study replicates results from a previous study that observed positive covariance in their respective SDG indicators [23]. However, the same study notes that the relationship between SDG 3 and 10 is negligible [23], whereas our analysis went on to highlight the salience of healthcare inequalities at this intersection [15, 19, 65]. Indeed, the NLP-powered analysis of unstructured text data offers opportunities for supplementary insights beyond the scope of existing indicators.

On the other hand, analogous to the divide between socioeconomic and environmental SDGs described by previous works [23, 87], we also observed that science connecting SDG 3-Health with SDGs 12-Consumption, 13-Climate, 14-Aquatic, and 15-Terrestrial is much less developed. This finding is remarkable considering the importance of this type of research – at the intersection between human health, socioeconomic issues, and environmental sustainability – for the One Health perspective, a central framework for science and policy on sustainability [27]. Future research into this persistent division is necessary if we are to address issues at the intersection of human and environmental health, including antibiotic resistance [38] and the transmission of zoonotic disease [12, 80].

Indeed, when addressing our second research question, our analysis offers insights on emergent research topics with the potential to bridge these divisions in the science of health and sustainability. Representing the relevant literature as a network of topics, we identified several ‘zipper themes’ which occupy central positions in the structure of health-sustainability research or can bridge significant gaps in this field. These are specific, actionable domains of research that cover a range of issues, from reproductive and maternal health to public transportation in developing cities, from climate change and schistosomiasis to environmental and health impacts of accidents in maritime transportation. In some cases, related bodies of literature on these themes were detected as distinct, unique topics by our models, revealing opportunities for new synergies in health and sustainability research, including within the One Health framework. As an example, insights from research on topic 158 (diffusion of schistosomiasis via freshwater snail migration) could promote scientific advances on topic 42 (treatment of schistosomiasis via praziquantel), although these are detected as separate topics in two different network communities (community 9 on infectious diseases and community 10 on wildlife ecology and zoonoses, respectively). Recognizing the interdependencies between climate and health sustainability, Pedersen (2014a; 2014b) empirically forecast increases in the incidence of intestinal schistosomiasis, induced by habitat loss, that will persist until 2055 [80, 81]. More recent studies have then added that biodiversity loss can have a similar impact on the transmission of zoonotic diseases [12, 88]. This most recent uptake of the climate change–zoonosis zipper theme that was being studied nearly a decade earlier validates our approach. Further applications could help guide research and policy to improve the preparedness of contemporary societies to future pandemics and public health emergencies.

## Conclusion

For almost a decade, health and sustainability scholars have advocated for the systematic synthesis of scientific literature and evidence, recognizing that the exponential growth of research volumes in this multidisciplinary field means that an increasing number of studies, insights and innovations risk to be overlooked or ignored [1, 2, 89]. Especially in research on complex topics such as global health, health systems, health inequalities, and sustainable development, literature and evidence syntheses are needed to appreciate the different aspects of multifaceted problems, recognize knowledge gaps, and learn lessons for future interventions [2]. The method proposed here illustrates each of these aims. The synthesis of large bodies of evidence on sustainability and health increasingly relies on scoping review methods, able to map and distill knowledge from hundreds of articles around the same substantive topic [4, 6, 90, 91]. In comparison, the combination of NLP and network science techniques presented here can map topics, connections, and gaps in science from much larger volumes of literature (nearly 30,000 articles in our case) around a more broadly defined topical area (here, health and sustainability). While our study does not match the nuance and detail of scoping reviews, it provides important information about the state and landscape of relevant research, including main topics, existing and missing connections between them, and promising directions for future work.

Moving forward, a major challenge will be to reposition and ‘rewire’ scientific efforts on SDGs 12-Consumption, 13-Climate, 14-Aquatic, and 15-Terrestrial on the map of health-sustainability research, increasing their substantive proximity to other goals in global science [87]. This would enable the identification of novel, interdisciplinary topics of research and policy that could connect and co-advance health with socioeconomic and environmental sustainability goals. Delving into the results of the current study, we identify several pairs of research topics that, through a One Health perspective, have the potential to produce – or are already in the process of producing – research, interventions, and technologies to co-advance multiple SDGs. These research topics represent promising paths forward toward the sustainable development goal to “ensure health lives and promote well-being for all at all ages” [92]. Approaching frontier research with this mindset would be a transformational starting point for scientists, funding agencies, and donors committed to developing interdisciplinary research to promote human health and well-being along with the other SDGs.

## Electronic supplementary material

Below is the link to the electronic supplementary material.

Supplementary Material 1

## Data Availability

Data were collected via the dimensions.ai application programming interface (API). A contractual agreement is required for access.

## Abbreviations

API: Application Programming Interface
DMNC: Density of maximum neighborhood component
Doc2vec: Document-to-Vector
HDBSCAN: Hierarchical density-based spatial clustering of applications with noise
IDE: Integrated Development Environment
NLP: Natural language processing
SDG: Sustainable development goal(s)
Top2vec: Topic-to-Vector
UMAP: Uniform manifold approximation and projection
UN: United nations
Word2vec: Word-to-Vector

## References

[1] Landhuis E (2016). Scientific literature: information overload. Nat 2016 5357612.

[2] Nature Sustainability. Editorial: Evidence synthesis for sustainability. Nat. Sustain. 2020 310 3, 771–771 (2020).

[3] Callaghan M et al. Machine-learning-based evidence and attribution mapping of 100,000 climate impact studies. *Nat. Clim. Chang* 2021 1111 11, 966–972 (2021).

[4] Piñeiro V et al. A scoping review on incentives for adoption of sustainable agricultural practices and their outcomes. *Nat. Sustain* 2020 310 3, 809–820 (2020).

[5] Ricciardi V et al. A scoping review of research funding for small-scale farmers in water scarce regions. *Nat. Sustain* 2020 310 3, 836–844 (2020).

[6] Stathers T et al. A scoping review of interventions for crop postharvest loss reduction in sub-Saharan Africa and South Asia. *Nat. Sustain* 2020 310 3, 821–835 (2020).

[7] Lavis JN, Posada FB, Haines PA, Osei E (2004). Use of research to inform public policymaking. Lancet.

[8] United Nations. *Transforming our world: the 2030 Agenda for Sustainable Development*. https://undocs.org/en/A/70/L.1 (2016).

[9] Biermann F (2022). Scientific evidence on the political impact of the Sustainable Development Goals. Nat Sustain 2022.

[10] Collins FS (2011). Reengineering Translational Science: the time is right. Sci Transl Med.

[11] Glasgow RE, Chambers D, Developing Robust (2012). Sustainable, implementation Systems using Rigorous, Rapid and relevant Science. Clin Transl Sci.

[12] Hemida MG, Ba Abduallah MM (2020). The SARS-CoV-2 outbreak from a one health perspective. One Heal.

[13] Adisasmito WB (2022). One health: a new definition for a sustainable and healthy future. PLOS Pathog.

[14] Sachs JD, Schmidt-Traub G, Lafortune G, Fuller G. The sustainable development goals and COVID-19: Sustainable Development Report 2020. (Cambridge University Press; 2020.

[15] Bambra C, Lynch J, Smith KE. *The unequal pandemic*. *The unequal pandemic*. Policy Press; 2021. 10.2307/J.CTV1QP9GNF.

[16] Sacco PL, De Domenico M (2021). Public health challenges and opportunities after COVID-19. Bull World Heal Organ.

[17] Alsan M, Chandra A, Simon K (2021). The great unequalizer: initial Health Effects of COVID-19 in the United States. J Econ Perspect.

[18] Tan AX, Hinman JA, Magid A, Nelson HS, Odden MC (2021). Association between Income Inequality and County-Level COVID-19 cases and deaths in the US. JAMA Netw Open.

[19] Blundell R (2022). Inequality and the COVID-19 Crisis in the United Kingdom. Annu Rev Econom.

[20] Arora S, Bhaukhandi KD, Mishra PK (2020). Coronavirus lockdown helped the environment to bounce back. Sci Total Environ.

[21] Bates AE (2021). Global COVID-19 lockdown highlights humans as both threats and custodians of the environment. Biol Conserv.

[22] de León EA (2021). Beyond building back better: imagining a future for human and planetary health. Lancet Planet Heal.

[23] De Neve JE, Sachs JD (2020). The SDGs and human well-being: a global analysis of synergies, trade-offs, and regional differences. Sci Rep 2020.

[24] Nilsson M, Griggs D, Visbeck M, Policy (2016). Map the interactions between Sustainable Development Goals. Nature.

[25] Le Blanc D (2015). Towards integration at last? The Sustainable Development Goals as a network of targets. Sustain Dev.

[26] Pradhan P, Costa L, Rybski D, Lucht W, Kropp JP (2017). A systematic study of sustainable development goal (SDG) interactions. Earth’s Futur.

[27] Nature E. Get the Sustainable Development Goals back on track. Nature https://www.nature.com/articles/d41586-019-03907-4 (2020).

[28] Collste D, Pedercini M, Cornell SE (2017). Policy coherence to achieve the SDGs: using integrated simulation models to assess effective policies. Sustain Sci.

[29] Pedercini M, Arquitt S, Collste D, Herren H (2019). Harvesting synergy from sustainable development goal interactions. Proc Natl Acad Sci U S A.

[30] Nilsson M, Griggs D, Visbeck M, Policy (2016). Map the interactions between Sustainable Development Goals. Nature.

[31] Lusseau D, Mancini F. Estimating the sustainome income-based variation in sustainable development goal interaction networks. 2, 242–7 (2019).

[32] Xu Z et al. Assessing progress towards sustainable development over space and time. Nature 577, (2020). 10.1038/s41586-019-1846-3 31894145

[33] Fuso Nerini F (2019). Connecting climate action with other Sustainable Development Goals. Nat Sustain.

[34] Pham-Truffert M, Metz F, Fischer M, Rueff H, Messerli P (2020). Interactions among Sustainable Development Goals: knowledge for identifying multipliers and virtuous cycles. Sustain Dev.

[35] Wang M et al. Accounting for interactions between Sustainable Development Goals is essential for water pollution control in China. *Nat. Commun* 2022 131 13, 1–13 (2022). 10.1038/s41467-022-28351-3 PMC8826988 35136079

[36] Chen Q (2021). Artificial Intelligence in Action: addressing the COVID-19 pandemic with Natural Language Processing. Annu Rev Biomed data Sci.

[37] Sachs JD, Kroll C, Lafortune G, Fuller G, Woelm F. The decade of action for the sustainable development goals: Sustainable Development Report 2021. Cambridge University Press; 2021.

[38] Hernando-Amado S, Coque TM, Baquero F, Martínez JL. Defining and combating antibiotic resistance from One Health and Global Health perspectives. *Nat. Microbiol* 2019 49 4, 1432–1442 (2019). 10.1038/s41564-019-0503-9 31439928

[39] Rudolph L, Caplan J, Ben-Moshe K, Dillon L. *Health in All Policies: A Guide for State and Local Governments*. https://stacks.cdc.gov/view/cdc/109299 (2013).

[40] G7. G7 Carbis Bay Health Declaration. https://www.gov.uk/government/publications/g7-carbis-bay-health-declaration (2021).

[41] G20. 2021 Declaration of the G20 Health Ministers. http://www.g20.utoronto.ca/2021/210906-health.html (2021).

[42] European Observatory on Health Systems and Policies &, McKee M. Drawing light from the pandemic: a new strategy for health and sustainable development - a review of the evidence. World Health Organizaation, Regional Office for Europe; 2021.

[43] Sachs JD, Lafortune G, Kroll C, Fuller G, Woelm F. From crisis to sustainable development: the SDGs as roadmap to 2030 and beyond - Sustainable Development Report 2022. Cambridge University Press; 2022.

[44] Porciello J, Ivanina M, Islam M, Einarson S, Hirsh H. Accelerating evidence-informed decision-making for the Sustainable Development Goals using machine learning. *Nat. Mach. Intell* 2020 210 2, 559–565 (2020).

[45] Digital, Science. *Contextualizing Sustainable Development Research: Using Dimensions to explore the global landscape of research on Sustainable Development Goals*. https://digitalscience.figshare.com/articles/report/Contextualizing_Sustainable_Development_Research/12200081 (2020) doi:10.6084/m9.gshare.12200081.

[46] R Core Team. R: A language and environment for statistical computing. Foundation for Statistical Computing, Vienna, Austria. https://www.R-project.org/ (2021).

[47] Van Rossum G, Drake FL. *Python 3 Reference Manual*. (CreateSpace, 2009).

[48] Csardi G, Nepusz T. The igraph software package for complex network research. InterJournal Complex Sy, 1695 (2006).

[49] Angelov D. Top2Vec: Distributed Representations of Topics. arXiv:2008.09470 (2020).

[50] Jalili M (2015). CentiServer: a Comprehensive Resource, web-based application and R Package for Centrality Analysis. PLoS ONE.

[51] Hook DW, Porter SJ, Herzog C, Dimensions (2018). Building context for search and evaluation. Front Res Metrics Anal.

[52] Singh VK, Singh P, Karmakar M, Leta J, Mayr P (2021). The journal coverage of web of Science, Scopus and Dimensions: a comparative analysis. Scientometrics.

[53] Mikolov T, Chen K, Corrado G, Dean J. Efficient estimation of word representations in vector space. in *1st International Conference on Learning Representations, ICLR 2013 - Workshop Track Proceedings* (International Conference on Learning Representations, ICLR, 2013).

[54] Le QV, Mikolov T. Distributed Representations of Sentences and Documents. in *31st International Conference on Machine Learning, ICML* 2014 vol. 4 2931–2939 (International Machine Learning Society (IMLS), 2014).

[55] McInnes L, Healy J, Melville JUMAP. Uniform Manifold Approximation and Projection for Dimension Reduction. arXiv:1802.03426 (2018).

[56] Campello RJGB, Moulavi D, Sander J. Density-Based Clustering Based on Hierarchical Density Estimates. in *Pacific-Asia Conference on Knowledge Dicovery and Data Mining, PAKDD 2013: Advances in Knowledge Discovery and Data Mining* 160–172 (Springer, Berlin, Heidelberg, 2013). doi:10.1007/978-3-642-37456-2_14.

[57] Fortunato S (2010). Community detection in graphs. Phys Rep.

[58] Pons P, Latapy M. Computing Communities in Large Networks Using Random Walks. in *ISCIS 2005: Computer and Information Sciences - ISCIS 2005* vol. 3733 LNCS 284–293 (Springer, Berlin, Heidelberg, 2005).

[59] Weitz N, Carlsen H, Nilsson M, Skånberg K (2018). Towards systemic and contextual priority setting for implementing the 2030 agenda. Sustain Sci.

[60] Allen C, Metternicht G, Wiedmann T, Prioritising (2019). SDG targets: assessing baselines, gaps and interlinkages. Sustain Sci.

[61] Asadikia A, Rajabifard A, Kalantari M (2021). Systematic prioritisation of SDGs: machine learning approach. World Dev.

[62] Borgatti SP, Everett MG. Three perpsectives on centrality. In: Light R, Moody J, editors. The Oxford Handbook of Social Networks. Oxford University Press; 2020.

[63] Lin CY (2008). Hubba: hub objects analyzer—a framework of interactome hubs identification for network biology. Nucleic Acids Res.

[64] Marchiori M, Latora V (2000). Harmony in the small-world. Phys A Stat Mech its Appl.

[65] Ali B, Chauhan S (2020). Inequalities in the utilisation of maternal health care in rural India: Evidences from National Family Health Survey III & IV. BMC Public Health.

[66] Pulok MH, Sabah MNU, Uddin J, Enemark U (2016). Progress in the utilization of antenatal and delivery care services in Bangladesh: where does the equity gap lie?. BMC Pregnancy Childbirth.

[67] Di Mascio P, Fusco G, Grappasonni G, Moretti L, Ragnoli A (2018). Geometrical and functional criteria as a Methodological Approach to implement a new cycle path in an existing Urban Road Network: a Case Study in Rome. Sustain 2018.

[68] Jacyna M, Wasiak M, Kłodawski M, Gołȩbiowski P (2017). Modelling of Bicycle Traffic in the Cities using VISUM. Procedia Eng.

[69] Stringhini S (2015). Decreasing educational differences in mortality over 40†years: evidence from the Turin Longitudinal Study (Italy). J Epidemiol Community Heal.

[70] Leclerc A, Chastang JF, Menvielle G, Luce D (2006). Socioeconomic inequalities in premature mortality in France: have they widened in recent decades?. Soc Sci Med.

[71] Roy K (2018). Assessment of under nutrition with composite index of anthropometric failure (CIAF) among under-five children in a rural area of West Bengal, India. Int J Contemp Pediatr.

[72] Tariku A (2016). Nearly half of preschool children are stunted in Dembia district, Northwest Ethiopia: a community based cross-sectional study. Arch Public Heal.

[73] Yilmaz F, Alp S, Yilmaz F, Alp S (2016). Underlying factors of Occupational Accidents: the case of Turkey. Open J Saf Sci Technol.

[74] García Mainar I, Montuenga Gómez V (2009). Causas de los accidentes de trabajo en España: análisis longitudinal con datos de panel. Gac Sanit.

[75] Hajizadeh M (2018). Socioeconomic inequalities in child vaccination in low/middle-income countries: what accounts for the differences?. J Epidemiol Community Health.

[76] Bobo FT, Hayen A (2020). Decomposition of socioeconomic inequalities in child vaccination in Ethiopia: results from the 2011 and 2016 demographic and health surveys. BMJ Open.

[77] Poggensee G (2005). A six-year follow-up of schoolchildren for urinary and intestinal schistosomiasis and soil-transmitted helminthiasis in Northern Tanzania. Acta Trop.

[78] Siza JE (2015). Prevalence of Schistosomes and Soil-Transmitted Helminths among Schoolchildren in Lake Victoria Basin, Tanzania. Korean J Parasitol.

[79] M’Bra RK et al. Risk factors for schistosomiasis in an urban area in northern Côte d’Ivoire. Infect Dis Poverty 7, (2018). 10.1186/s40249-018-0431-6 PMC5958400 29773076

[80] Pedersen UB (2014). Modelling spatial distribution of snails transmitting parasitic worms with importance to human and animal health and analysis of distributional changes in relation to climate. Geospat Health.

[81] Pedersen UB (2014). Modelling climate change impact on the spatial distribution of fresh water snails hosting trematodes in Zimbabwe. Parasites and Vectors.

[82] Short SE, Goldberg RE (2015). Children living with HIV-Infected adults: estimates for 23 countries in sub-saharan Africa. PLoS ONE.

[83] Wete AT, Zerfu TA, Anbese AT. Magnitude and associated factors of wasting among under five orphans in Dilla town, southern Ethiopia: 2018: a cross-sectional study. BMC Nutr. 5, (2019). 10.1186/s40795-019-0295-6 PMC7050719 32153946

[84] Eliopoulou E, Papanikolaou A, Diamantis P, Hamann R. Analysis of tanker casualties after the Oil Pollution Act (USA, 1990). *Proc. Inst. Mech. Eng. Part M J. Eng. Marit. Environ* 226, 301–312 (2012) doi:10.1371/journal.pone.0142580.

[85] Uğurlu Ö, Köse E, Yıldırım U, Yüksekyıldız E (2015). Marine accident analysis for collision and grounding in oil tanker using FTA method. Marit Policy Manag.

[86] Mueller N, Westerby M, Nieuwenhuijsen M. Health impact assessments of shipping and port-sourced air pollution on a global scale: a scoping literature review. Environ Res. 2022;114460. 10.1016/J.ENVRES.2022.114460. 10.1016/j.envres.2022.114460 36191619

[87] Smith TB, Vacca R, Mantegazza L, Capua I (2021). Natural language processing and network analysis provide novel insights on policy and scientific discourse around Sustainable Development Goals. Sci Rep 2021.

[88] Keesing F, Ostfeld RS. Impacts of biodiversity and biodiversity loss on zoonotic diseases. *Proc. Natl. Acad. Sci. U. S. A* 118, e2023540118 (2021). 10.1073/pnas.2023540118 PMC8092607 33820825

[89] McKinnon MC, Cheng SH, Garside R, Masuda YJ, Miller DC (2015). Sustainability: Map the evidence. Nat 2015 5287581.

[90] Bunge AC, Wood A, Halloran A, Gordon LJ (2022). A systematic scoping review of the sustainability of vertical farming, plant-based alternatives, food delivery services and blockchain in food systems. Nat Food 2022.

[91] Lut I, Harron K, Hardelid P, O’Brien M, Woodman J (2021). Linking fathers and children in administrative data for public health research: a systematic scoping review. Lancet.

[92] Sweileh WM (2020). Bibliometric analysis of scientific publications on ‘sustainable development goals’ with emphasis on ‘good health and well-being’ goal (2015–2019). Global Health.

