# Supplementary material for: Discovering new pathways toward integration between health and sustainable development goals with natural language processing and network science
Source: Global Health. 2023 Jun 29;19:44. doi: 10.1186/s12992-023-00943-8 (PMC10311734; doi:10.1186/s12992-023-00943-8)
Supplement: Supplementary file 1 — Supplementary Material 1 [file 12992_2023_943_MOESM1_ESM.docx]

**Supplementary Materials**

Discovering new pathways toward integration between health and sustainable development goals with a synthesis of natural language processing and network science

**Note on the intersections between SDG literature**

The volume of research relevant to each of the SDGs varies substantially. Of the 5,256,652 publications indexed in dimensions.ai as relevant to one or more of the goals, the vast majority concern SDG 7-Energy (25.9%; N = 1,362,871) and 3-Health (21.7%; N = 1,141,895), followed by 16-Peace (12.3%; N = 648,651), 4-Education (10.6%; N = 559,122), and 13-Climate (8.8%; N = 464,048). Accordingly, there is also variation in the number of intersections between the SDGs (see Figures S1 and S2), here referring to co-assignation of an SDG tag in the dimensions.ai database ^1^. For instance, the largest intersection between SDGs is observed between 7-Energy and 13-Climate (N = 142,524, 46.5% of the 306,386 publications at the intersection of at least two SDGs). On the other hand, intersection between 5-Gender and 9-Industry or 5-Gender and 13-Climate is virtually nonexistent, with a respective 3 and 6 publications sharing these pairs of SDG tags. Surprisingly, despite its central role in sustainability, SDG 3-Health only accounts for 9.7% of all intersections between the goals – another motivating factor for selecting 3-Health as our focus.

**Data Preprocessing**

All abstracts were preprocessed and parsed in accordance with normal procedures (tokenization, lemmatization, part-of-speech tagging) using the spaCy python library. Preprocessed abstracts were reassembled, retaining lemmatized words with a Pennsylvania treebank part-of-speech tag indicative of a propensity to contain pertinent information ^2^^[[1]](#footnote-1)^. Stopwords (e.g., a, I, me, my, we, because, so) were omitted regardless of their part-of-speech.

**The Curse of Dimensionality**

Generating 300-dimensional embeddings for each of 64,575 words and 27,928 documents produces a dataset that is too high-dimensional for our subsequent analyses, with nearly 30 million cells in the combined dataset. To resolve this issue, UMAP is applied to reduce the dimension of embeddings assigned to each document and word, allowing cluster analysis of the 5-dimensional embedding representations ^3^.

**Network Centrality**

We use three centrality measures in this study: (1) Betweenness; (2) Harmonic Closeness; (3) Density of the Maximum Neighborhood Component (DMNC). Betweenness centrality identifies vertices located on the most geodesic (shortest) paths between the other vertices in the network – i.e. middle nodes, stations for information, or bridges between separate regions of the network ^4^. Topics with high betweenness gather concepts and language features with potential semantic links with diverse and separate areas of the literature. Harmonic centrality is the harmonic mean of all shortest weighted paths from a given topic to all other topics ^5^. Highly harmonic topics can be interpreted as having the highest overall proximity to all other topics in the network. Finally, DMNC identifies topics at the intersection of dense, highly-connected clusters or communities of topics ^6^, such as those identified here via the walktrap algorithm (see next section). DMNC is defined as the edge count, $E$, over node count, $N$, raised to the ɛ power ($E/N^{\varepsilon}$ with ɛ typically set to 1.67), for subgraph $MNC(v)$, the maximum connected component of the first neighborhood of the $v^{th}$ node omitting node $v$ itself. DMNC was developed to remedy the tendency of centrality measures to prioritize nodes with a large number of incident edges, and is able to identify ‘hubs’ (i.e. highly between nodes) with a smaller number of incident edges ^7^. Intuitively, DMNC can be interpreted as a localized betweenness measure, identifying topics at the intersection of broader themes in the corpus – rather than the universal intersection offered by betweenness.

**Junk topics**

Topics 101, 141, and 162 are ‘junk topics’, consisting of a small selection of abstracts (N = 202; 0.72% of our sample) containing hyperlinks and other forms of raw html formatting in lieu of substantive content. By extension, community 18 – which we have omitted from Table S1 – is a ‘junk community’, consisting of topics 141 and 162. These topics and community represent a subset of abstracts which circumvented our data filtering procedures. Due to the unique structure and formatting of the text data in these errant abstracts, top2vec filtered these topics and placed them towards the edge of the topical distribution presented in Figure 2. This ensures they have no impact on the structure of the network presented in Figure 3. The annotated network presented in Figure S4 showcases their isolation from the network and, by extension, topic model.

**Bibliography**

1. Digital Science *et al.* *Contextualizing Sustainable Development Research: Using Dimensions to explore the global landscape of research on Sustainable Development Goals*. https://digitalscience.figshare.com/articles/report/Contextualizing_Sustainable_Development_Research/12200081 (2020) doi:10.6084/m9..gshare.12200081.

2. Taylor, A., Marcus, M. & Santorini, B. The Penn Treebank: An Overview. in *Treebanks: Building and Using Parsed Corpora* (ed. Abeillé, A.) vol. 20 5–22 (Springer Netherlands, 2003).

3. Angelov, D. Top2Vec: Distributed Representations of Topics. arXiv:2008.09470 (2020).

4. Borgatti, S. P. & Everett, M. G. Three perpsectives on centrality. in *The Oxford Handbook of Social Networks* (eds. Light, R. & Moody, J.) (Oxford University Press, 2020).

5. Marchiori, M. & Latora, V. Harmony in the small-world. *Phys. A Stat. Mech. its Appl.* **285**, 539–546 (2000).

6. Lin, C. Y. *et al.* Hubba: hub objects analyzer—a framework of interactome hubs identification for network biology. *Nucleic Acids Res.* **36**, W438 (2008).

7. Chin, C. H. *et al.* cytoHubba: Identifying hub objects and sub-networks from complex interactome. *BMC Syst. Biol.* **8**, 1–7 (2014).


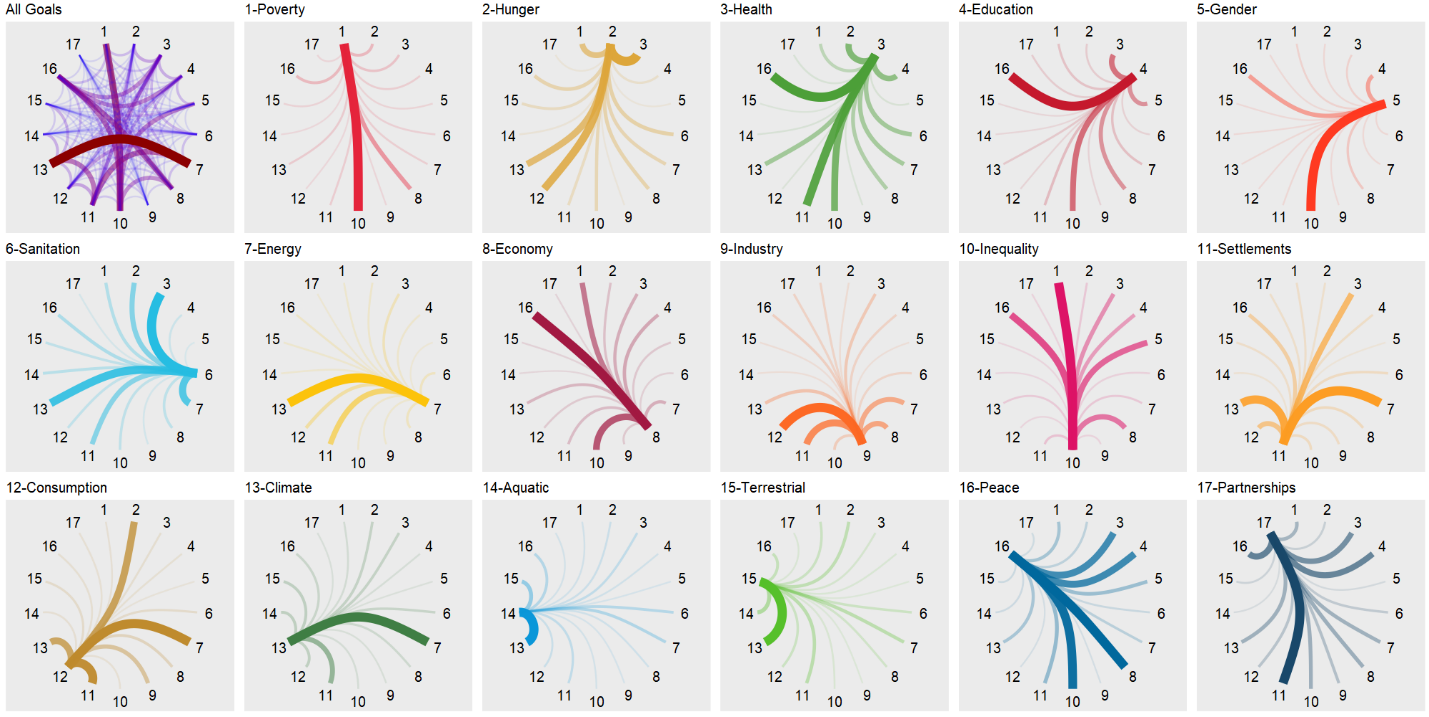


Figure S1. Intersection between the SDGs across all research indexed by dimensions.ai: counts of publications.


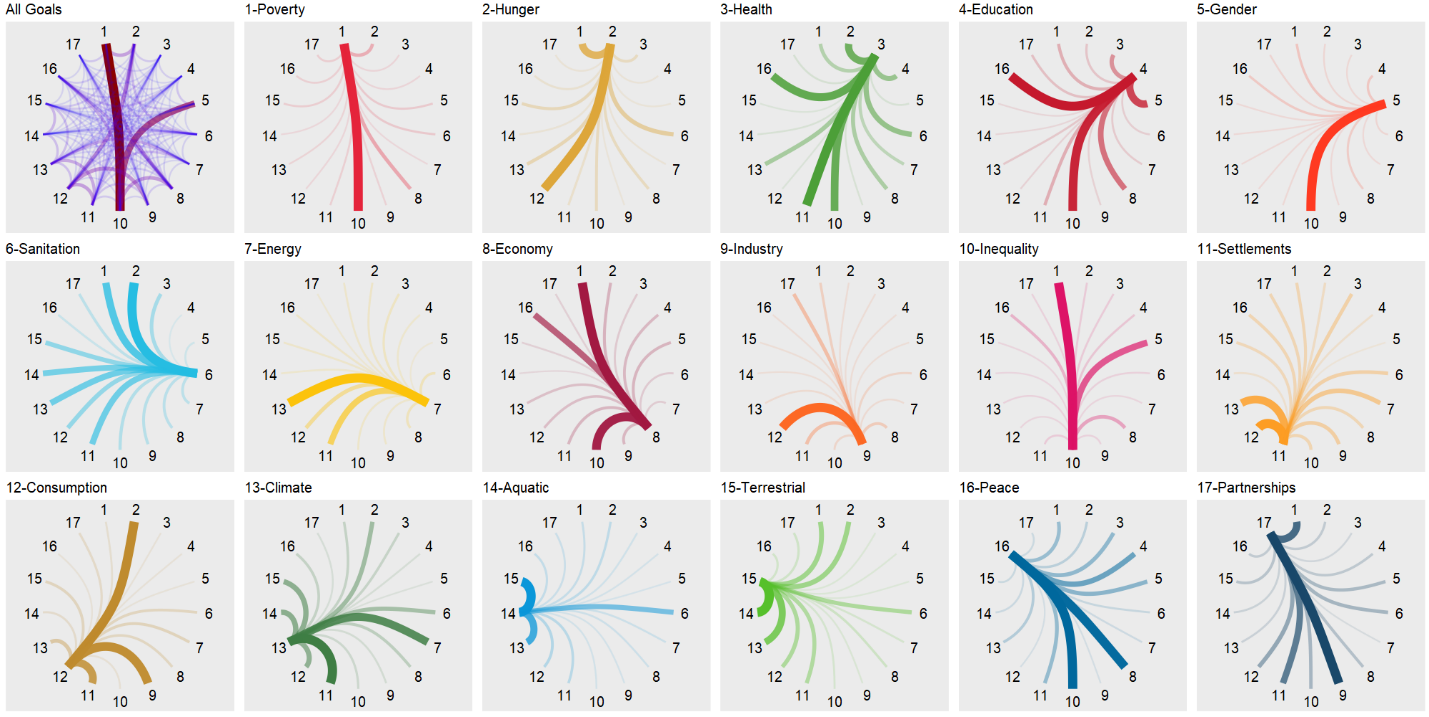


Figure S2. Intersection between the SDGs across all research indexed by dimensions.ai: Jaccard index (N publications with both SDG tags divided by N publications with either SDG tag).


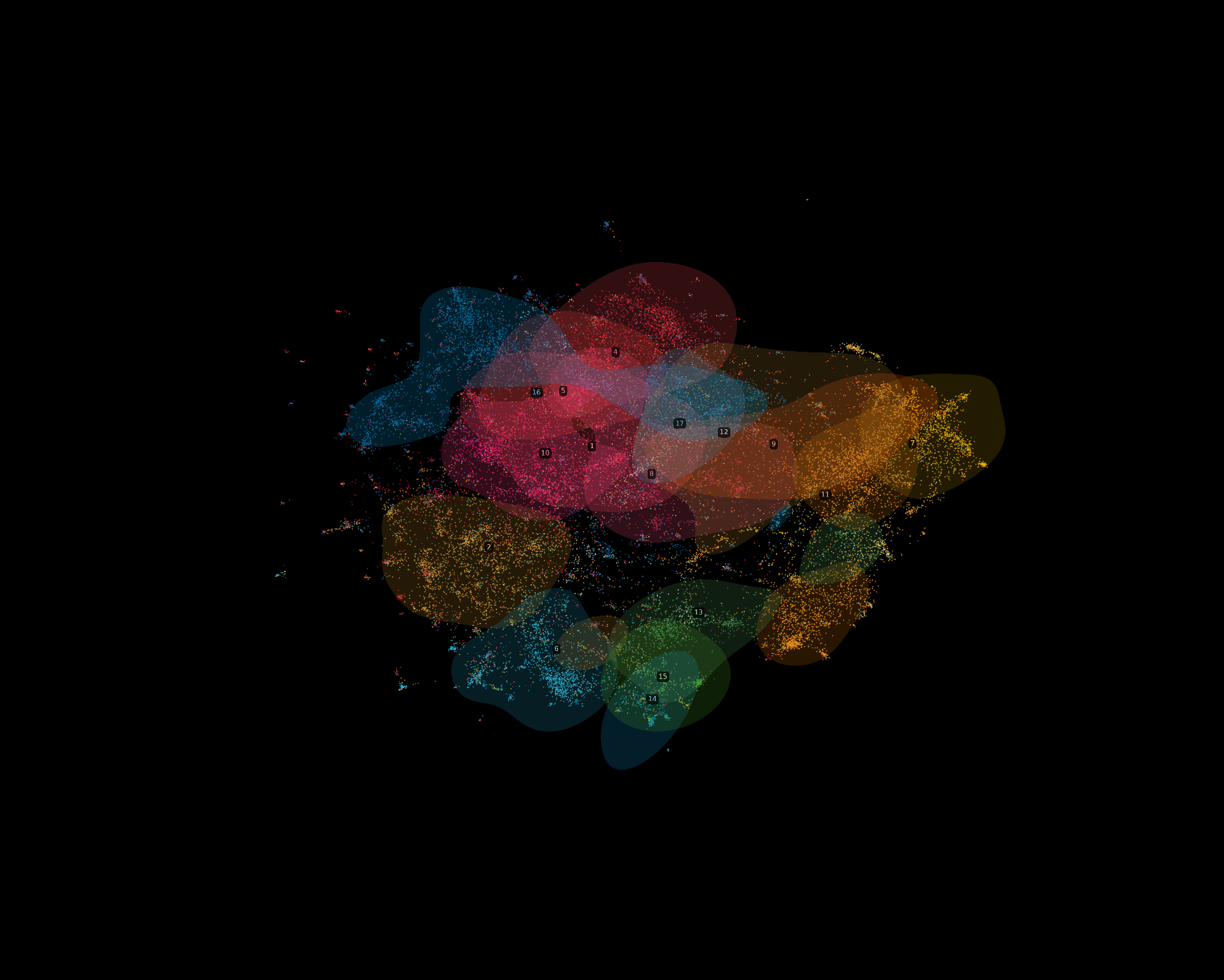


Figure S3. Map of all peer-reviewed publications classified by dimensions.ai as relevant to 3-Health and at least one other SDG. K-density used to outline regions of the map where most research into each SDG are located.


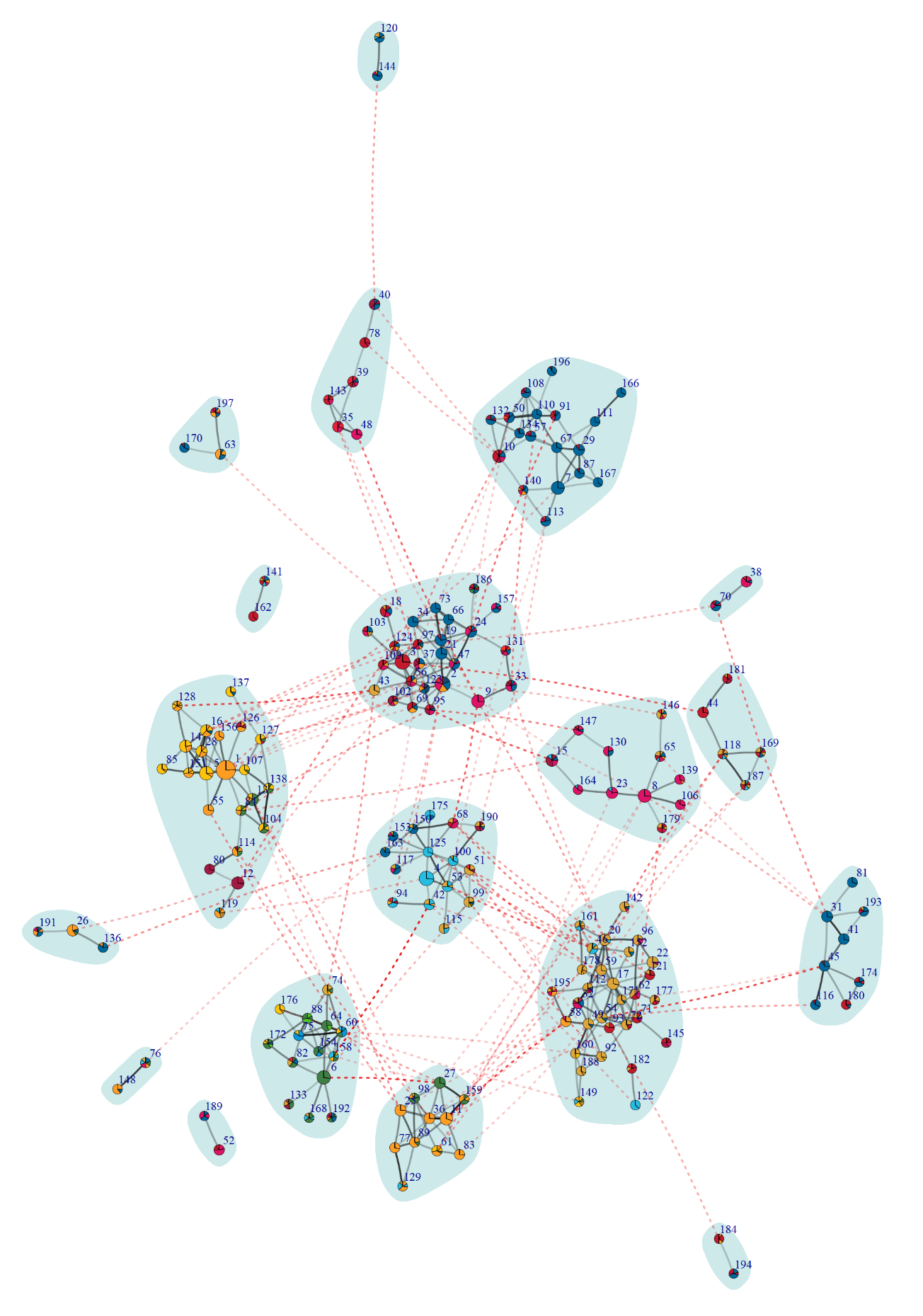


Figure S4. Annotated weighted network of cosine similarities between the topics discovered by top2vec. Graph rotated 90-degrees clockwise to better accommodate labels.


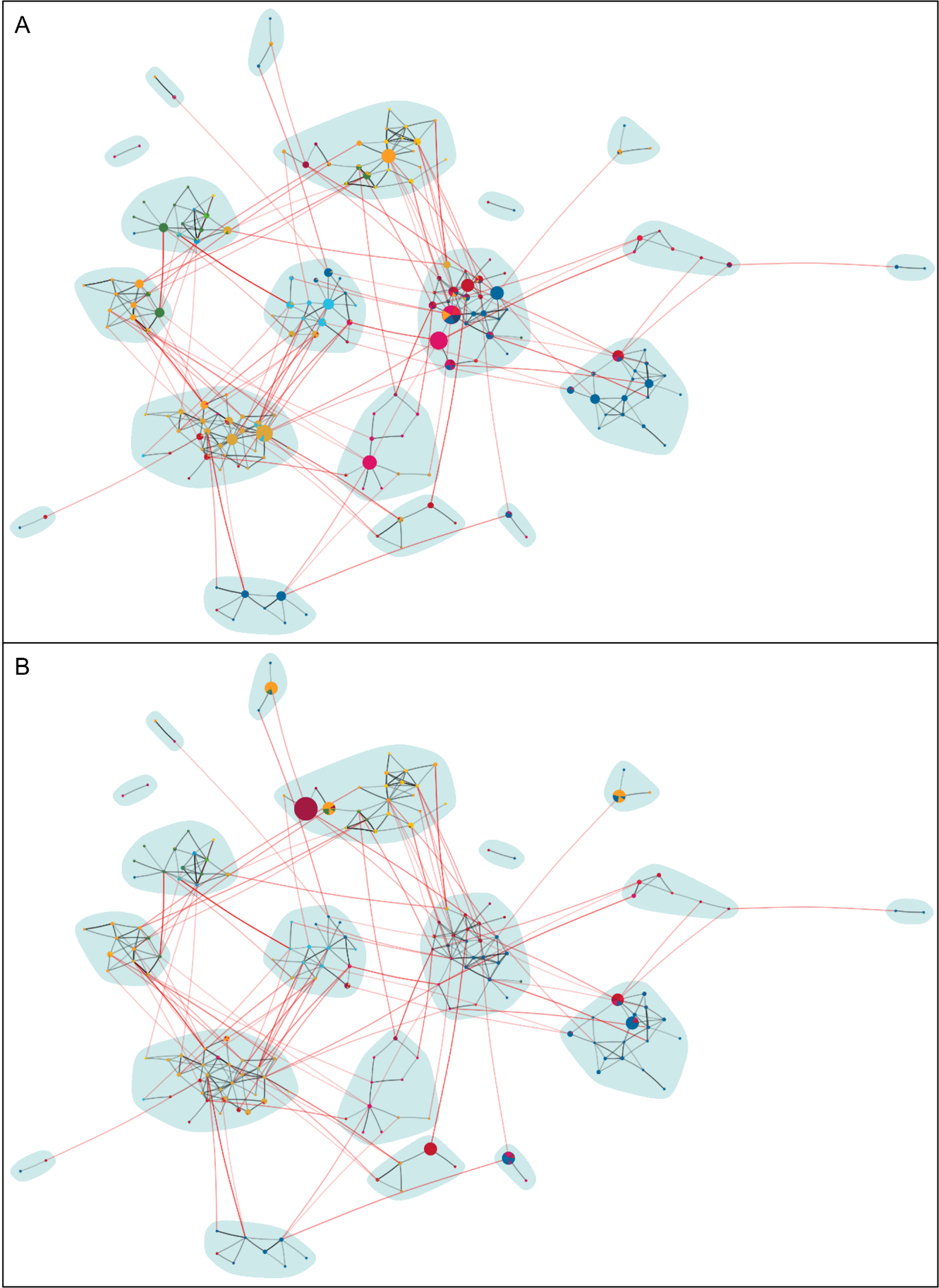


Figure S5. Weighted network of cosine similarities. Node colors represent the secondary SDGs paired with 3-Health. Node size is scaled to represent either A: Betweenness, or B: DMNC.

Table S1. Broader themes identified via walktrap community detection

| Community | Concept | # Topics |
| --- | --- | --- |
|  |  |  |
| 1 | Mental and physical health in offending and at-risk populations | 18 |
| 2 | Health disparities based on socioeconomic inequities | 11 |
| 3 | Technology, infrastructure, and the workplace | 22 |
| 4 | Emergency medicine and surgery | 8 |
| 5 | Healthcare access | 6 |
| 6 | Non-communicable diseases and conditions | 30 |
| 7 | Malnutrition in Southeast Asia | 5 |
| 8 | Health policies, politics, and ethics | 26 |
| 9 | Infectious diseases | 16 |
| 10 | Wildlife ecology and zoonoses | 14 |
| 11 | Emergency / disaster management and response | 3 |
| 12 | Environmental health | 11 |
| 13 | Behavioral disorders | 2 |
| 14 | Race and racism | 2 |
| 15 | Coronavirus and COVID-19 | 2 |
| 16 | Displaced people | 2 |
| 17 | Road safety and traffic accidents | 3 |
| 19 | Health disparities in breast, cervical, and colorectal cancers | 2 |
|  |  |  |

Community 18 represents an individual network component consisting only of topics 141 and 162,
both of which have been identified as ‘junk topics’ and have been removed from this table.

Table S3. Topic Descriptions

| **Topic** | **N** | **Community** | **Topic Words (Word Score)** |
| --- | --- | --- | --- |
| 7 | 488 | 1 | offender (0.367); correctional (0.341); probation (0.273); criminal (0.266); delinquent (0.266); abuser (0.256); offense (0.251); incarcerate (0.25); juvenile (0.249); jail (0.249) |
| 10 | 434 |  | transcribe (0.448); transcript (0.425); thematic (0.411); barrier (0.367); fgds (0.363); depth (0.361); qualitative (0.355); audio (0.343); informant (0.341); semi (0.335) |
| 29 | 239 |  | internalize (0.493); aggression (0.355); father (0.346); esteem (0.323); parenting (0.286); style (0.261); aggressive (0.253); emotional (0.248); mediate (0.24); relationship (0.238) |
| 50 | 167 |  | srh (0.566); srhr (0.352); sexually (0.33); menstruation (0.328); transmit (0.318); reproductive (0.31); contraception (0.308); sti (0.302); sexual (0.302); sexuality (0.301) |
| 57 | 153 |  | bisexual (0.627); transgender (0.625); lesbian (0.615); gay (0.601); lgbt (0.565); heterosexual (0.509); msm (0.487); trans (0.423); identity (0.334); stigma (0.31) |
| 67 | 134 |  | perpetration (0.573); victimization (0.485); cyber (0.343); date (0.335); ipv (0.287); abusive (0.26); teen (0.245); perpetrator (0.23); aggression (0.226); lifetime (0.214) |
| 87 | 105 |  | ideation (0.527); suicidal (0.498); maltreatment (0.368); attempt (0.365); suicide (0.297); thought (0.251); sedentary (0.243); internalize (0.237); eating (0.234); personality (0.221) |
| 91 | 102 |  | teenager (0.568); teenage (0.51); sexuality (0.415); sexual (0.285); girl (0.262); adolescent (0.259); sexually (0.254); representation (0.245); contraception (0.233); gendered (0.227) |
| 110 | 82 |  | rc (0.555); coercion (0.51); gbv (0.452); unintended (0.445); ipv (0.409); contraceptive (0.393); intimate (0.317); contraception (0.309); partner (0.276); pill (0.263) |
| 108 | 82 |  | prep (0.751); agyw (0.597); fsw (0.585); condom (0.449); sti (0.448); msm (0.333); prophylaxis (0.329); sexually (0.325); hiv (0.31); testing (0.26) |
| 111 | 81 |  | ptsd (0.695); posttraumatic (0.617); traumatic (0.437); dsm (0.365); stress (0.344); veteran (0.286); depression (0.27); internalize (0.263); disorder (0.26); event (0.254) |
| 113 | 80 |  | hcv (0.714); pwid (0.608); injection (0.488); inject (0.398); hepatitis (0.33); needle (0.299); substitution (0.283); viral (0.259); homelessness (0.256); exchange (0.239) |
| 132 | 69 |  | trafficking (0.614); obstetrician (0.377); survivor (0.29); gynecology (0.244); exploitation (0.231); gbv (0.229); restaurant (0.227); exploit (0.206); informed (0.201); clinician (0.2) |
| 134 | 66 |  | disclose (0.61); disclosure (0.424); confidentiality (0.276); ipv (0.243); intimate (0.227); privacy (0.227); partner (0.198); survivor (0.189); ask (0.187); screening (0.186) |
| 140 | 63 |  | homeless (0.728); homelessness (0.634); housing (0.31); shelter (0.276); progression (0.218); infestation (0.19); pwid (0.189); unstable (0.189); precarious (0.184); nhs (0.183) |
| 166 | 42 |  | veteran (0.71); army (0.472); gulf (0.436); military (0.425); posttraumatic (0.37); ptsd (0.316); va (0.28); personnel (0.253); civilian (0.25); deploy (0.247) |
| 167 | 42 |  | ace (0.806); aces (0.78); adversity (0.4); divorce (0.258); binge (0.251); adulthood (0.249); cumulative (0.24); dysfunction (0.236); childhood (0.231); adverse (0.218) |
| 196 | 27 |  | dv (0.805); hcp (0.238); picu (0.223); master (0.223); recession (0.195); seriously (0.187); domestic (0.184); ask (0.183); voluntary (0.178); mandatory (0.165) |
| 8 | 485 | 2 | ingestion (0.194); entirely (0.19); cause (0.188); baltic (0.183); census (0.18); scotland (0.177); intentional (0.174); fishery (0.173); circulatory (0.172); intoxication (0.168) |
| 15 | 347 |  | capita (0.424); causality (0.41); inflation (0.391); gdp (0.39); recession (0.355); macroeconomic (0.334); unemployment (0.318); gross (0.311); ordinary (0.31); econometric (0.307) |
| 23 | 272 |  | brazilian (0.397); municipality (0.379); brazil (0.356); northeast (0.343); autocorrelation (0.332); imr (0.285); schooling (0.254); unified (0.25); northeastern (0.243); color (0.243) |
| 65 | 141 |  | daly (0.63); gbd (0.57); yld (0.566); sdi (0.543); ui (0.512); burden (0.508); yll (0.477); standardise (0.393); uncertainty (0.364); lose (0.358) |
| 106 | 85 |  | sep (0.685); gradient (0.241); position (0.237); mediate (0.217); paternal (0.207); magnitude (0.202); cannabis (0.2); adjustment (0.199); rome (0.195); partially (0.195) |
| 130 | 69 |  | sus (0.726); unified (0.58); brazilian (0.512); brazil (0.41); municipality (0.345); municipal (0.316); de (0.312); janeiro (0.245); gerais (0.236); federal (0.236) |
| 139 | 64 |  | maori (0.729); nz (0.533); zealand (0.52); pacific (0.371); table (0.225); indigenous (0.216); ethnic (0.215); recognise (0.192); utilise (0.192); culturally (0.191) |
| 147 | 60 |  | austerity (0.783); recession (0.474); fiscal (0.408); crisis (0.408); deterioration (0.291); greece (0.274); subsequent (0.224); radical (0.223); deepen (0.215); articulate (0.214) |
| 146 | 60 |  | melinda (0.714); gates (0.692); bill (0.596); foundation (0.539); grant (0.36); funding (0.35); declaration (0.331); statement (0.322); approval (0.32); interpretation (0.298) |
| 164 | 45 |  | hdi (0.703); pearson (0.294); correlation (0.282); prostate (0.255); incidence (0.238); coefficient (0.236); gini (0.236); uruguay (0.226); pc (0.218); lung (0.218) |
| 179 | 38 |  | dementia (0.693); geriatric (0.228); elder (0.227); cognitive (0.21); id (0.193); hr (0.184); memory (0.183); scottish (0.176); working (0.173); adl (0.17) |
| 1 | 1277 | 3 | walking (0.257); sustainable (0.226); walk (0.217); intermodal (0.215); walkability (0.212); modal (0.209); planning (0.201); cycling (0.183); motorized (0.182); choice (0.18) |
| 5 | 564 |  | steering (0.384); maneuver (0.344); brake (0.339); controller (0.314); braking (0.305); motion (0.284); angle (0.277); stability (0.276); lateral (0.263); comfort (0.258) |
| 12 | 406 |  | occupational (0.451); workplace (0.404); employee (0.376); worker (0.356); job (0.356); ohs (0.316); ppe (0.291); working (0.277); work (0.27); osh (0.267) |
| 13 | 367 |  | cng (0.39); diesel (0.317); ghg (0.292); gasoline (0.29); biodiesel (0.268); euro (0.267); duty (0.264); fleet (0.246); nox (0.246); biofuel (0.241) |
| 14 | 361 |  | convolutional (0.51); neural (0.488); cnn (0.484); dnn (0.4); deep (0.382); bit (0.371); accuracy (0.369); memory (0.368); hardware (0.361); automatically (0.36) |
| 16 | 344 |  | cloud (0.526); blockchain (0.503); iot (0.468); iov (0.416); server (0.406); secure (0.401); privacy (0.401); thing (0.393); computing (0.356); overhead (0.344) |
| 28 | 243 |  | vanets (0.611); packet (0.568); vanet (0.553); slot (0.438); wireless (0.42); topology (0.415); ad (0.391); mac (0.378); communication (0.378); routing (0.375) |
| 55 | 158 |  | db (0.538); noise (0.507); acoustic (0.469); sound (0.456); pavement (0.225); hear (0.221); asphalt (0.195); permissible (0.192); hearing (0.191); circle (0.189) |
| 80 | 111 |  | billion (0.553); productivity (0.512); discount (0.463); lose (0.44); cost (0.429); loss (0.428); valuation (0.377); indirect (0.371); dollar (0.32); premature (0.32) |
| 84 | 106 |  | rebound (0.559); elasticity (0.344); tax (0.342); taxis (0.323); price (0.314); taxation (0.311); gasoline (0.308); econometric (0.295); pricing (0.262); macroeconomic (0.254) |
| 85 | 106 |  | deformation (0.471); absorb (0.423); frontal (0.419); absorption (0.382); occupant (0.382); crush (0.354); box (0.35); crash (0.315); finite (0.314); interior (0.26) |
| 104 | 87 |  | biodiesel (0.679); biofuel (0.616); ethanol (0.542); blend (0.496); renewable (0.389); diesel (0.344); bio (0.34); gasoline (0.32); fossil (0.295); fuel (0.286) |
| 107 | 83 |  | ev (0.601); electric (0.321); electrification (0.267); charge (0.24); battery (0.236); electricity (0.211); penetration (0.211); discount (0.209); pricing (0.186); powertrain (0.185) |
| 114 | 79 |  | valuation (0.672); willingness (0.437); monetary (0.409); pay (0.309); discount (0.299); cost (0.293); billion (0.261); hypothetical (0.259); pine (0.255); lose (0.248) |
| 119 | 76 |  | waste (0.584); disposal (0.544); recycling (0.391); segregation (0.35); hazardous (0.346); solid (0.32); reuse (0.276); biomedical (0.268); handling (0.249); corporation (0.248) |
| 126 | 70 |  | tourism (0.69); tourist (0.648); destination (0.35); visitor (0.283); traveler (0.279); resort (0.263); revolution (0.253); marketing (0.242); era (0.223); travel (0.221) |
| 127 | 69 |  | electrification (0.553); electricity (0.446); grid (0.388); renewable (0.288); generation (0.265); solar (0.257); ambitious (0.257); mini (0.244); pv (0.228); sector (0.215) |
| 128 | 69 |  | uav (0.686); unmanned (0.599); aerial (0.589); drone (0.556); image (0.441); camera (0.381); flight (0.378); sensing (0.364); satellite (0.305); remote (0.29) |
| 137 | 64 |  | nuclear (0.616); radiation (0.318); fatality (0.239); accident (0.234); electricity (0.221); es (0.214); casualty (0.208); coal (0.203); oecd (0.201); severe (0.191) |
| 138 | 64 |  | hydrogen (0.563); biofuel (0.393); biodiesel (0.345); renewable (0.344); blend (0.304); gasoline (0.293); diesel (0.279); cng (0.258); fossil (0.256); prospectively (0.253) |
| 151 | 56 |  | rfid (0.761); tag (0.399); reader (0.386); radio (0.365); smart (0.313); send (0.304); iot (0.288); wireless (0.278); vanets (0.266); read (0.259) |
| 156 | 51 |  | tram (0.736); stop (0.253); complicate (0.191); operator (0.189); railway (0.184); std (0.182); crossing (0.181); tribal (0.181); satisfied (0.18); workload (0.179) |
| 31 | 224 | 4 | drowning (0.499); drown (0.458); unintentional (0.439); poisoning (0.438); fatal (0.376); injury (0.352); intentional (0.352); homicide (0.344); intent (0.317); undetermined (0.307) |
| 41 | 188 |  | autopsy (0.538); coroner (0.474); homicidal (0.465); drown (0.464); unnatural (0.417); manner (0.399); forensic (0.392); undetermined (0.387); accidental (0.373); drowning (0.371) |
| 45 | 171 |  | wound (0.484); penetrate (0.479); gunshot (0.471); amputation (0.449); extremity (0.444); neck (0.397); thoracic (0.379); explosive (0.376); abdominal (0.369); surgery (0.365) |
| 81 | 109 |  | binge (0.463); drinker (0.436); cirrhosis (0.434); alcohol (0.421); alcoholic (0.373); liver (0.367); consumption (0.343); drink (0.337); beverage (0.298); foetal (0.293) |
| 116 | 78 |  | maxillofacial (0.652); mandibular (0.621); fracture (0.587); facial (0.583); bone (0.404); nasal (0.362); sport (0.344); interpersonal (0.312); soft (0.306); etiology (0.302) |
| 174 | 40 |  | tbi (0.729); brain (0.35); traumatic (0.277); sci (0.259); injury (0.252); picu (0.233); penetrate (0.211); motor (0.2); injure (0.198); np (0.198) |
| 180 | 37 |  | spinal (0.625); sci (0.576); cord (0.496); neurological (0.233); yld (0.23); palsy (0.229); wheelchair (0.22); tbi (0.217); amputation (0.216); traumatic (0.205) |
| 193 | 31 |  | sids (0.7); sudden (0.483); postneonatal (0.328); unexpected (0.314); sleep (0.288); certify (0.251); certification (0.244); coroner (0.242); asphyxia (0.241); transform (0.236) |
| 35 | 208 | 5 | catastrophic (0.539); impoverishment (0.502); payment (0.497); pocket (0.491); oop (0.483); expenditure (0.372); push (0.316); expense (0.314); hardship (0.313); financial (0.298) |
| 39 | 192 |  | aca (0.575); medicaid (0.559); uninsured (0.481); expansion (0.426); medicare (0.414); insurance (0.347); insure (0.336); affordable (0.319); federal (0.3); eligibility (0.299) |
| 40 | 189 |  | asylum (0.674); seeker (0.664); undocumented (0.59); refugee (0.499); immigration (0.461); migrant (0.437); immigrant (0.388); arrive (0.374); migration (0.354); arrival (0.298) |
| 48 | 169 |  | horizontal (0.475); pro (0.342); inpatient (0.338); che (0.326); hi (0.32); outpatient (0.315); rich (0.303); decomposition (0.282); decompose (0.279); utilization (0.254) |
| 78 | 116 |  | lep (0.563); proficiency (0.524); interpreter (0.452); english (0.441); language (0.404); speak (0.374); latino (0.319); immigrant (0.306); asian (0.276); california (0.261) |
| 143 | 61 |  | nhis (0.647); enrolment (0.487); scheme (0.282); ghana (0.25); insure (0.238); utilisation (0.233); rsby (0.232); fee (0.221); premium (0.21); card (0.21) |
| 17 | 302 | 6 | sam (0.301); antiretroviral (0.3); art (0.294); heu (0.272); ahr (0.241); kaplan (0.228); pmtct (0.226); hiv (0.222); count (0.22); uninfected (0.218) |
| 20 | 285 |  | stunt (0.374); stunting (0.348); underweight (0.348); haz (0.306); height (0.258); stunted (0.256); waste (0.249); wasting (0.245); feeding (0.238); anthropometric (0.236) |
| 22 | 280 |  | sugar (0.514); fruit (0.484); vegetable (0.483); fat (0.465); diet (0.448); beverage (0.404); intake (0.4); dietary (0.394); cereal (0.388); food (0.377) |
| 32 | 217 |  | eclampsia (0.501); haemorrhage (0.475); rupture (0.447); puerperal (0.43); obstruct (0.429); uterine (0.401); hemorrhage (0.387); obstetric (0.386); placenta (0.38); obstetrics (0.367) |
| 46 | 171 |  | ors (0.5); rehydration (0.455); rotavirus (0.414); dehydration (0.321); diarrhoea (0.286); zinc (0.272); diarrhoeal (0.269); diarrhea (0.264); vaccine (0.25); gastroenteritis (0.24) |
| 49 | 167 |  | placental (0.574); fetal (0.511); epigenetic (0.504); iugr (0.476); intrauterine (0.461); fetus (0.458); offspring (0.456); endocrine (0.391); placenta (0.388); hypoxia (0.381) |
| 54 | 158 |  | postoperative (0.517); operative (0.464); closure (0.425); parenteral (0.411); surgery (0.395); surgical (0.378); obstruction (0.374); repair (0.361); fistula (0.335); elective (0.272) |
| 58 | 152 |  | trimester (0.467); preterm (0.442); gestational (0.39); singleton (0.381); sga (0.309); birthweight (0.309); gestation (0.296); birth (0.29); lbw (0.289); fetal (0.276) |
| 59 | 149 |  | iron (0.508); folic (0.407); deficiency (0.39); micronutrient (0.388); zinc (0.368); supplementation (0.339); vitamin (0.311); supplement (0.277); anemia (0.26); hemoglobin (0.236) |
| 62 | 147 |  | diabetes (0.502); mellitus (0.471); diabetic (0.448); diabete (0.431); insulin (0.417); dm (0.407); glucose (0.396); pressure (0.371); hypertension (0.333); cholesterol (0.328) |
| 71 | 126 |  | frail (0.449); frailty (0.426); geriatric (0.425); adl (0.406); multimorbidity (0.296); functional (0.274); malignant (0.261); musculoskeletal (0.256); accumulation (0.237); malformation (0.237) |
| 72 | 125 |  | dialysis (0.572); esrd (0.545); transplantation (0.524); transplant (0.474); kidney (0.469); ckd (0.467); renal (0.455); hd (0.393); albumin (0.351); crp (0.264) |
| 92 | 102 |  | mouse (0.515); cytokine (0.467); il (0.383); immune (0.347); expression (0.341); antigen (0.333); cell (0.301); proliferation (0.301); receptor (0.298); inflammatory (0.291) |
| 93 | 100 |  | palsy (0.575); cp (0.486); cerebral (0.472); hearing (0.442); impairment (0.361); developmental (0.346); hear (0.303); speech (0.294); seizure (0.245); ohs (0.241) |
| 96 | 98 |  | overweight (0.509); obesity (0.42); obese (0.365); bmi (0.324); underweight (0.291); sedentary (0.271); ncd (0.253); height (0.219); communicable (0.219); stunted (0.214) |
| 112 | 81 |  | lbw (0.394); baby (0.362); gestational (0.294); birth (0.274); trimester (0.272); preterm (0.267); weight (0.263); eclampsia (0.254); parity (0.248); singleton (0.243) |
| 121 | 73 |  | leprosy (0.66); nerve (0.39); foot (0.298); grade (0.273); reaction (0.245); disability (0.245); sensory (0.232); mycobacterium (0.196); multidrug (0.194); occurrence (0.19) |
| 122 | 72 |  | trachoma (0.703); tf (0.617); lga (0.541); tt (0.507); cleanliness (0.441); facial (0.379); certify (0.317); mapping (0.282); elimination (0.266); blindness (0.26) |
| 142 | 62 |  | insecure (0.611); insecurity (0.525); secure (0.344); food (0.301); security (0.268); fi (0.21); canadian (0.193); approximate (0.185); marginal (0.176); deforestation (0.169) |
| 145 | 60 |  | pension (0.729); sick (0.308); sickness (0.264); retirement (0.258); sweden (0.242); entitlement (0.239); hazard (0.233); germany (0.227); leave (0.216); german (0.212) |
| 149 | 59 |  | broiler (0.683); poultry (0.562); bird (0.428); carcass (0.42); feed (0.346); trait (0.302); probiotic (0.295); conversion (0.284); meat (0.283); ne (0.263) |
| 152 | 54 |  | va (0.655); verbal (0.431); autopsy (0.327); asphyxia (0.281); deceased (0.279); prematurity (0.273); notify (0.268); assign (0.244); leading (0.223); gastroenteritis (0.214) |
| 160 | 46 |  | pup (0.701); rat (0.508); offspring (0.415); wean (0.346); gestation (0.332); placental (0.307); litter (0.299); oxidative (0.269); ewe (0.267); expression (0.248) |
| 161 | 46 |  | ari (0.735); cough (0.292); breathing (0.253); cooking (0.252); zero (0.252); overcrowding (0.249); alri (0.212); media (0.205); pneumonia (0.2); stove (0.196) |
| 171 | 41 |  | picu (0.723); shock (0.316); intensive (0.315); pediatric (0.293); scoring (0.267); mechanical (0.266); admit (0.264); critically (0.254); ventilation (0.252); paediatric (0.235) |
| 177 | 39 |  | scd (0.727); sickle (0.65); transfusion (0.488); cell (0.298); blood (0.247); thalassemia (0.227); readmission (0.227); carrier (0.223); premium (0.222); hb (0.213) |
| 178 | 39 |  | alri (0.754); rsv (0.352); ari (0.29); rainy (0.268); biomass (0.264); overcrowding (0.26); season (0.254); pneumonia (0.229); seasonal (0.224); bias (0.216) |
| 182 | 36 |  | blindness (0.757); eye (0.552); visual (0.529); blind (0.47); vision (0.439); impairment (0.367); trachoma (0.357); bilateral (0.339); avoidable (0.266); onchocerciasis (0.256) |
| 188 | 33 |  | microbiota (0.782); gut (0.66); probiotic (0.323); microbe (0.277); alter (0.258); phenotype (0.255); composition (0.254); responder (0.251); pup (0.248); altered (0.234) |
| 195 | 31 |  | ptb (0.727); lbw (0.4); sputum (0.255); investigator (0.244); preterm (0.243); sga (0.232); wuhan (0.224); tb (0.21); iugr (0.2); gestational (0.199) |
| 44 | 176 | 7 | pretest (0.422); junior (0.312); quasi (0.301); attitude (0.268); experimental (0.258); knowledge (0.228); experiment (0.222); behavior (0.209); student (0.198); sampling (0.197) |
| 118 | 76 |  | toddler (0.625); puskesmas (0.481); regency (0.366); exclusive (0.291); kesehatan (0.288); dengan (0.286); sampling (0.27); indonesian (0.269); untuk (0.268); indonesia (0.268) |
| 169 | 42 |  | untuk (0.788); dan (0.777); hasil (0.777); pada (0.772); yang (0.769); penelitian (0.764); dengan (0.758); kesehatan (0.715); di (0.654); puskesmas (0.502) |
| 181 | 36 |  | kap (0.629); attitude (0.313); governorate (0.261); needle (0.248); misconception (0.237); df (0.234); thalassemia (0.233); fiji (0.229); ckd (0.215); dm (0.209) |
| 187 | 33 |  | correspondence (0.558); java (0.457); doi (0.39); email (0.385); ir (0.382); indonesia (0.327); https (0.323); keyword (0.302); puskesmas (0.289); kesehatan (0.257) |
| 2 | 768 | 8 | sdgs (0.493); sdg (0.449); mdgs (0.437); mdg (0.419); sustainable (0.412); goal (0.411); millennium (0.403); agenda (0.393); development (0.365); sustainability (0.359) |
| 3 | 746 |  | graduate (0.259); learner (0.256); learning (0.24); faculty (0.239); teacher (0.232); competency (0.221); curriculum (0.221); undergraduate (0.217); postgraduate (0.213); learn (0.209) |
| 9 | 462 |  | pnc (0.387); attendant (0.323); sba (0.322); anc (0.318); skilled (0.315); delivery (0.305); antenatal (0.277); caste (0.265); postnatal (0.245); utilization (0.244) |
| 18 | 301 |  | cinahl (0.596); embase (0.595); medline (0.553); search (0.532); cochrane (0.526); scopus (0.524); systematic (0.517); pubmed (0.504); prospero (0.498); psycinfo (0.473) |
| 19 | 286 |  | beneficence (0.56); ethical (0.545); bioethic (0.53); ethic (0.51); autonomy (0.421); moral (0.412); principle (0.409); dilemma (0.391); informed (0.332); obligation (0.316) |
| 21 | 280 |  | fctc (0.564); wto (0.5); treaty (0.467); convention (0.446); trade (0.443); agreement (0.44); patent (0.436); ip (0.353); intellectual (0.344); negotiation (0.326) |
| 24 | 262 |  | feminist (0.538); gendered (0.512); discourse (0.453); sexuality (0.416); ethnographic (0.361); politic (0.352); activist (0.348); motherhood (0.335); meaning (0.333); neoliberal (0.32) |
| 33 | 215 |  | wife (0.566); husband (0.564); married (0.469); contraceptive (0.432); contraception (0.4); spouse (0.395); couple (0.378); marriage (0.37); fertility (0.364); spousal (0.338) |
| 34 | 210 |  | liability (0.397); offense (0.343); medan (0.341); sanction (0.324); negligence (0.321); law (0.278); offence (0.267); punishment (0.266); officer (0.264); police (0.262) |
| 37 | 198 |  | ukraine (0.574); ukrainian (0.499); russian (0.473); federation (0.419); sphere (0.396); russia (0.378); modernization (0.36); soviet (0.329); digitalization (0.327); territory (0.315) |
| 43 | 178 |  | agriculture (0.554); crop (0.49); farming (0.454); precision (0.411); farmer (0.41); yield (0.387); agricultural (0.36); irrigation (0.343); fertilizer (0.335); weed (0.327) |
| 47 | 170 |  | neoliberal (0.572); globalization (0.47); politic (0.386); discourse (0.36); political (0.333); bioethic (0.317); actor (0.303); transnational (0.302); essay (0.293); articulate (0.29) |
| 56 | 153 |  | digitalization (0.618); transformation (0.453); digital (0.392); economy (0.365); revolution (0.341); technological (0.328); innovation (0.297); competitive (0.294); era (0.287); internet (0.284) |
| 66 | 137 |  | constitutional (0.596); constitution (0.566); court (0.401); amendment (0.38); judicial (0.376); right (0.364); litigation (0.364); supreme (0.332); fundamental (0.331); substantive (0.328) |
| 69 | 132 |  | conference (0.536); proceeding (0.471); book (0.421); forum (0.397); engineering (0.39); chair (0.352); professor (0.344); talk (0.313); reader (0.298); topic (0.295) |
| 73 | 125 |  | conscience (0.739); conscientious (0.687); objection (0.662); denial (0.412); freedom (0.403); liberty (0.4); moral (0.392); church (0.39); refusal (0.382); refuse (0.379) |
| 95 | 99 |  | fiscal (0.522); debt (0.506); budget (0.38); finance (0.36); macroeconomic (0.347); financing (0.346); deficit (0.326); revenue (0.313); recession (0.307); spending (0.301) |
| 97 | 97 |  | mhealth (0.628); ehealth (0.555); phone (0.413); smartphone (0.41); mobile (0.356); internet (0.316); ubiquitous (0.295); technology (0.291); app (0.286); interested (0.281) |
| 102 | 90 |  | enterprise (0.399); financing (0.367); finance (0.352); sized (0.249); micro (0.223); informal (0.213); financial (0.204); payment (0.197); institution (0.196); reform (0.196) |
| 103 | 89 |  | pharmacist (0.716); pharmacy (0.508); pharmaceutical (0.353); medication (0.31); prescribe (0.271); prescribed (0.252); inefficiency (0.244); prescription (0.234); medicine (0.226); shortage (0.214) |
| 109 | 82 |  | telemedicine (0.733); botswana (0.282); telehealth (0.27); technology (0.259); sustainability (0.25); digitalization (0.239); networking (0.237); ict (0.228); technological (0.219); reimbursement (0.214) |
| 124 | 71 |  | ai (0.667); intelligence (0.446); artificial (0.43); machine (0.34); big (0.315); transformation (0.281); computational (0.28); analytic (0.277); customer (0.277); fashion (0.271) |
| 123 | 71 |  | ppp (0.785); private (0.359); partnership (0.304); tribal (0.271); procurement (0.251); sustainability (0.242); scheme (0.239); governance (0.239); project (0.229); sector (0.228) |
| 131 | 69 |  | son (0.688); daughter (0.501); preference (0.45); fertility (0.305); selective (0.291); father (0.241); logit (0.233); imbalance (0.229); marriage (0.227); want (0.217) |
| 157 | 51 |  | aboriginal (0.7); indigenous (0.305); colonization (0.252); australian (0.248); colonial (0.246); racism (0.226); culturally (0.216); remote (0.215); shame (0.207); root (0.204) |
| 186 | 33 |  | hansen (0.761); colonial (0.291); fault (0.233); politic (0.231); japanese (0.23); chair (0.225); bioethic (0.223); dr (0.223); professor (0.22); newspaper (0.213) |
| 4 | 686 | 9 | physicochemical (0.253); bacteriological (0.215); watershed (0.202); revenue (0.199); microbiological (0.199); fluctuation (0.19); velocity (0.188); pedal (0.184); absorb (0.183); waterborne (0.181) |
| 42 | 180 |  | schistosoma (0.579); schistosomiasis (0.56); praziquantel (0.543); mansoni (0.54); haematobium (0.525); helminth (0.451); snail (0.439); sth (0.433); phs (0.407); mda (0.403) |
| 51 | 166 |  | tb (0.584); tuberculosis (0.539); sputum (0.467); mdr (0.452); mycobacterium (0.44); bcg (0.412); multidrug (0.358); resistant (0.334); pulmonary (0.286); contact (0.281) |
| 53 | 158 |  | typhi (0.508); shigellosis (0.466); typhoid (0.441); enteric (0.347); salmonella (0.319); conjugate (0.308); strain (0.29); resistant (0.284); shigella (0.283); vaccine (0.28) |
| 68 | 132 |  | pertussis (0.556); diphtheria (0.548); immunisation (0.517); tetanus (0.485); immunization (0.443); vaccination (0.438); polio (0.408); measle (0.382); immunize (0.372); vaccine (0.354) |
| 94 | 100 |  | lf (0.636); ntd (0.628); lymphatic (0.627); filariasis (0.624); onchocerciasis (0.568); neglected (0.411); elimination (0.404); mda (0.363); sth (0.351); roadmap (0.322) |
| 99 | 97 |  | leishmaniasis (0.674); visceral (0.663); vl (0.644); leishmania (0.6); cutaneous (0.496); sand (0.332); cl (0.3); sudan (0.285); fly (0.271); parasitic (0.25) |
| 100 | 96 |  | hav (0.657); hev (0.62); seroprevalence (0.478); hepatitis (0.448); antibody (0.371); elisa (0.365); genotype (0.312); endemicity (0.307); igg (0.305); virus (0.278) |
| 115 | 79 |  | scabie (0.669); infestation (0.546); lesion (0.333); skin (0.304); cutaneous (0.227); carie (0.211); unusual (0.207); overcrowding (0.203); worm (0.203); fungal (0.195) |
| 117 | 78 |  | sierra (0.625); ebola (0.621); leone (0.621); liberia (0.559); outbreak (0.281); multisectoral (0.259); lf (0.255); guinea (0.248); mistrust (0.237); coordinate (0.223) |
| 125 | 71 |  | cholera (0.507); vaccine (0.36); outbreak (0.303); herd (0.294); vaccination (0.291); campaign (0.284); epidemic (0.262); dose (0.252); ebola (0.24); vaccinate (0.236) |
| 150 | 56 |  | polio (0.733); eradication (0.494); immunization (0.42); eradicate (0.403); measle (0.4); campaign (0.383); vaccine (0.381); vaccination (0.372); diphtheria (0.342); vaccinate (0.342) |
| 153 | 54 |  | drc (0.679); congo (0.434); democratic (0.404); humanitarian (0.345); ebola (0.303); conflict (0.293); republic (0.269); zone (0.267); geo (0.256); armed (0.239) |
| 163 | 45 |  | smallpox (0.744); terrorism (0.319); vaccination (0.303); vaccine (0.272); diphtheria (0.272); terrorist (0.257); influenza (0.242); twentieth (0.232); lac (0.229); measle (0.227) |
| 175 | 40 |  | dracunculiasis (0.771); eradication (0.561); worm (0.443); eradicate (0.348); guinea (0.323); mali (0.321); sudan (0.263); assembly (0.247); elimination (0.216); unicef (0.204) |
| 190 | 32 |  | hbv (0.726); hepatitis (0.397); antibody (0.303); antigen (0.296); elisa (0.289); vaccination (0.283); hav (0.261); vaccinate (0.258); hev (0.257); seroprevalence (0.252) |
| 6 | 550 | 10 | chikungunya (0.329); vector (0.315); dengue (0.31); arbovirus (0.285); infectious (0.267); arthropod (0.265); nile (0.248); aedes (0.242); albopictus (0.238); aegypti (0.235) |
| 60 | 148 |  | mussel (0.472); larvae (0.342); recruitment (0.31); juvenile (0.309); larval (0.308); predation (0.288); spawn (0.278); sediment (0.27); dispersal (0.263); reef (0.256) |
| 64 | 143 |  | stand (0.501); tree (0.497); pine (0.484); forest (0.455); seedling (0.422); drought (0.359); plot (0.355); stem (0.337); disturbance (0.276); ha (0.27) |
| 74 | 123 |  | fungus (0.436); pest (0.413); plant (0.327); pesticide (0.303); crop (0.276); fungal (0.274); rotation (0.272); seed (0.266); medicinal (0.261); colonization (0.261) |
| 75 | 122 |  | fishery (0.505); fishing (0.461); spawn (0.45); salmon (0.429); catch (0.417); maturity (0.389); exploitation (0.358); length (0.347); fish (0.332); recruitment (0.324) |
| 82 | 108 |  | trypanosomiasis (0.624); cattle (0.528); livestock (0.52); hat (0.486); veterinary (0.479); zoonosis (0.42); wildlife (0.414); animal (0.4); zoonotic (0.354); dog (0.33) |
| 88 | 104 |  | mammal (0.498); conservation (0.447); wildlife (0.418); extinction (0.406); endanger (0.303); wild (0.293); predation (0.286); biodiversity (0.285); habitat (0.265); fragmented (0.253) |
| 133 | 68 |  | falciparum (0.782); vivax (0.774); plasmodium (0.761); malaria (0.483); insecticide (0.317); export (0.268); suitability (0.252); parasite (0.241); net (0.238); elimination (0.234) |
| 154 | 53 |  | bleaching (0.738); coral (0.724); reef (0.675); colony (0.312); stressor (0.298); hypoxia (0.274); recover (0.268); shallow (0.263); bay (0.257); surprisingly (0.252) |
| 158 | 49 |  | snail (0.654); schistosomiasis (0.468); schistosoma (0.446); mansoni (0.407); dam (0.347); intermediate (0.337); haematobium (0.335); praziquantel (0.31); freshwater (0.301); habitat (0.3) |
| 168 | 42 |  | dhf (0.746); dengue (0.427); df (0.412); hemorrhagic (0.402); conducive (0.29); fever (0.288); jakarta (0.276); mosquito (0.244); regency (0.237); monthly (0.235) |
| 172 | 40 |  | rabie (0.707); dog (0.65); bat (0.566); bite (0.318); conservation (0.285); wildlife (0.274); owner (0.273); zoonotic (0.258); principally (0.255); animal (0.232) |
| 176 | 39 |  | turbine (0.642); bird (0.428); carcass (0.4); wind (0.397); farm (0.373); collision (0.308); bat (0.288); strike (0.256); extinction (0.236); wildlife (0.23) |
| 192 | 32 |  | zikv (0.759); zika (0.615); arbovirus (0.357); chikungunya (0.323); asymptomatic (0.291); aedes (0.283); virus (0.271); dengue (0.265); aegypti (0.253); epidemic (0.253) |
| 26 | 259 | 11 | disaster (0.674); earthquake (0.591); preparedness (0.498); tsunami (0.488); emergency (0.398); evacuation (0.379); rescue (0.371); responder (0.359); hurricane (0.35); flood (0.347) |
| 136 | 65 |  | terrorist (0.604); terrorism (0.506); attack (0.423); explosive (0.276); disaster (0.259); casualty (0.258); preparedness (0.257); threat (0.239); cyber (0.231); surge (0.224) |
| 191 | 32 |  | ems (0.766); prehospital (0.542); emergency (0.305); limb (0.238); responder (0.228); render (0.215); rescue (0.205); definitive (0.203); arrival (0.198); triage (0.197) |
| 11 | 418 | 12 | spline (0.365); microm (0.332); gam (0.272); crossover (0.225); series (0.225); lag (0.218); freedom (0.215); microg (0.214); pool (0.206); daily (0.2) |
| 25 | 262 |  | morning (0.289); ultrafine (0.241); bc (0.236); rush (0.234); near (0.199); clinic (0.193); roadside (0.177); unprotected (0.172); site (0.167); tailor (0.166) |
| 27 | 255 |  | heat (0.387); rcp (0.339); wave (0.321); adaptation (0.287); heatwave (0.283); projection (0.282); extreme (0.247); cold (0.21); future (0.203); veteran (0.188) |
| 36 | 205 |  | tehran (0.316); iranian (0.254); italian (0.185); iran (0.181); rcp (0.18); microm (0.173); megacitie (0.162); lose (0.16); save (0.16); industrialize (0.159) |
| 61 | 147 |  | cooking (0.575); stove (0.534); biomass (0.486); wood (0.448); heating (0.432); coal (0.425); indoor (0.407); burning (0.404); cook (0.398); burn (0.394) |
| 77 | 116 |  | pahs (0.639); aromatic (0.503); metal (0.414); hydrocarbon (0.411); carcinogenic (0.376); copper (0.344); ng (0.293); toxic (0.269); deposition (0.258); sediment (0.246) |
| 83 | 108 |  | allergic (0.645); asthma (0.456); allergy (0.375); exacerbation (0.366); airway (0.27); epidemiologic (0.257); mechanistic (0.241); detrimental (0.226); trap (0.216); implicate (0.201) |
| 89 | 103 |  | sulfate (0.478); dust (0.357); nitrate (0.349); aerosol (0.344); mineral (0.332); ammonia (0.297); particle (0.261); combustion (0.26); constituent (0.258); ion (0.257) |
| 98 | 97 |  | bc (0.413); oc (0.384); methane (0.377); aerosol (0.349); precursor (0.326); anthropogenic (0.293); halve (0.251); premature (0.238); globally (0.214); chemistry (0.208) |
| 129 | 69 |  | pb (0.591); cu (0.556); zn (0.544); metal (0.522); cr (0.427); fe (0.38); hg (0.351); carcinogenic (0.342); cd (0.318); ni (0.312) |
| 159 | 48 |  | percentile (0.433); spline (0.31); th (0.305); cold (0.293); temperature (0.26); hot (0.25); freedom (0.239); nonlinear (0.223); heat (0.203); rcp (0.202) |
| 184 | 35 | 13 | asd (0.783); autism (0.677); developmental (0.382); spectrum (0.352); pc (0.284); adhd (0.222); palsy (0.196); causal (0.191); radar (0.182); confound (0.178) |
| 194 | 31 |  | adhd (0.699); ps (0.396); deficit (0.344); autism (0.286); puerperal (0.234); urgency (0.22); absenteeism (0.184); asd (0.183); comorbidity (0.18); attention (0.174) |
| 38 | 193 | 14 | segregation (0.576); racial (0.426); racism (0.32); race (0.308); black (0.28); white (0.269); color (0.261); structural (0.226); minority (0.22); american (0.205) |
| 70 | 128 |  | alaska (0.614); ai (0.499); native (0.434); white (0.389); american (0.374); racial (0.367); indian (0.357); black (0.332); hispanic (0.313); ethnic (0.31) |
| 76 | 118 | 15 | cov (0.768); sars (0.736); coronavirus (0.716); covid (0.635); pandemic (0.59); lockdown (0.423); spread (0.331); deadly (0.314); ebola (0.264); globe (0.256) |
| 148 | 59 |  | lockdown (0.76); covid (0.572); coronavirus (0.461); pandemic (0.459); cov (0.415); sars (0.374); aqi (0.336); april (0.281); march (0.252); spread (0.248) |
| 120 | 74 | 16 | idp (0.746); internally (0.696); displace (0.675); displacement (0.587); camp (0.517); refugee (0.376); humanitarian (0.338); syrian (0.236); sphere (0.23); un (0.224) |
| 144 | 61 |  | syrian (0.713); syria (0.536); lebanon (0.523); refugee (0.521); displace (0.419); camp (0.399); displacement (0.348); internally (0.348); turkey (0.348); jordan (0.325) |
| 63 | 146 | 17 | helmet (0.635); seatbelt (0.587); speeding (0.557); motorcycle (0.481); rider (0.474); seat (0.428); wear (0.423); motorcyclist (0.411); restraint (0.405); belt (0.372) |
| 170 | 41 |  | yrbss (0.79); drunk (0.49); inactivity (0.451); behavior (0.402); intercourse (0.39); fruit (0.378); precede (0.375); unhealthy (0.359); ride (0.358); std (0.357) |
| 197 | 26 |  | rti (0.821); motorcyclist (0.373); motorcycle (0.27); seatbelt (0.262); helmet (0.252); unskilled (0.229); df (0.229); consciousness (0.222); injury (0.214); bicyclist (0.209) |
| 141 | 62 | 18 | dx (0.399); doi (0.397); journal (0.35); ethics (0.348); vol (0.341); approval (0.288); higher (0.27); org (0.26); cite (0.246); http (0.242) |
| 162 | 46 |  | commons (0.656); unrestricted (0.603); creative (0.59); attribution (0.586); noncommercial (0.552); properly (0.551); cite (0.541); nc (0.525); reproduction (0.51); commercial (0.502) |
| 52 | 164 | 19 | cervical (0.487); cancer (0.454); hpv (0.436); mammography (0.376); screening (0.373); colorectal (0.363); breast (0.346); prostate (0.34); pap (0.336); screen (0.245) |
| 189 | 33 |  | crc (0.81); colorectal (0.387); adoption (0.212); county (0.211); racial (0.207); screening (0.188); hard (0.187); cancer (0.171); ev (0.17); normalize (0.17) |
| 30 | 227 | Isolates | smoker (0.603); cigarette (0.584); smoke (0.569); tobacco (0.554); quit (0.551); smoking (0.544); nicotine (0.492); cessation (0.434); ban (0.416); smokeless (0.41) |
| 79 | 114 |  | vessel (0.539); ship (0.534); spill (0.505); maritime (0.466); tanker (0.439); shipping (0.433); marine (0.364); oil (0.336); port (0.302); cargo (0.289) |
| 86 | 105 |  | dental (0.631); dentist (0.616); carie (0.579); tooth (0.519); oral (0.489); dentistry (0.419); maxillofacial (0.225); viewpoint (0.222); restoration (0.219); appointment (0.171) |
| 90 | 102 |  | crispr (0.724); editing (0.682); cas (0.658); genome (0.591); edit (0.523); genetic (0.445); embryo (0.43); gene (0.428); engineering (0.391); genomic (0.355) |
| 101 | 94 |  | xsi (0.805); xmlschema (0.797); jats (0.791); nlm (0.782); ncbi (0.734); xmlns (0.719); nih (0.707); instance (0.701); www (0.67); http (0.611) |
| 105 | 86 |  | georgia (0.486); republic (0.477); guyana (0.463); mauritius (0.457); oceania (0.436); venezuela (0.434); peru (0.434); sao (0.421); cuba (0.412); guinea (0.406) |
| 135 | 66 |  | colombian (0.701); colombia (0.497); peace (0.366); armed (0.357); affiliate (0.217); municipality (0.206); conflict (0.203); war (0.189); humanitarian (0.181); logistical (0.179) |
| 155 | 51 |  | chw (0.68); farmworker (0.575); northwest (0.237); participatory (0.212); conducive (0.206); seasonal (0.202); favorable (0.198); equitable (0.192); accountable (0.18); community (0.178) |
| 165 | 45 |  | saudi (0.615); arabia (0.61); rta (0.603); game (0.305); kingdom (0.251); happen (0.192); informal (0.19); terrorism (0.185); limb (0.181); leisure (0.18) |
| 173 | 40 |  | orphan (0.726); extended (0.307); arrangement (0.246); worsen (0.222); nearby (0.222); paternal (0.194); fgds (0.183); incentive (0.18); emotion (0.179); judicial (0.179) |
| 183 | 36 |  | roma (0.818); serbia (0.382); mediator (0.346); croatia (0.269); romania (0.266); activist (0.265); bp (0.23); bulgaria (0.228); czech (0.226); yr (0.218) |
| 185 | 35 |  | nlmcategory (0.832); abstracttext (0.81); label (0.676); results (0.425); amp (0.421); aims (0.41); conclusions (0.398); the (0.37); methods (0.37); cent (0.302) |

1. Nouns (NN, NNP, NNS, NNPS), prepositions (IN), verbs (VB, VBD, VBG, VBN, VBP, VBZ), adverbs (RB, RBR, RBS, WRB), determiners (DT), adjectives (JJ, JJR, JJS), pronouns (PRP, WP), coordinating conjunctions (CC), cardinal numbers (CD), interjections (UH), and symbols (SYM). [↑](#footnote-ref-1)
